# Optically pumped colloidal-quantum-dot lasing in LED-like devices with an integrated optical cavity

**DOI:** 10.1038/s41467-019-14014-3

**Published:** 2020-01-14

**Authors:** Jeongkyun Roh, Young-Shin Park, Jaehoon Lim, Victor I. Klimov

**Affiliations:** 1 Chemistry Division, Los Alamos National Laboratory, Los Alamos, NM 87545 USA; 2 Centre for High Technology Materials, University of New Mexico, Albuquerque, NM 87131 USA; 3 Department of Chemical Engineering and Department of Energy System Research, Ajou University, Suwon, 16499 Republic of Korea; 4 Present Address: Department of Electrical Engineering, Pusan National University, Busan, 46241 Republic of Korea

**Keywords:** Nanoscale devices, Lasers, LEDs and light sources, Quantum dots

## Abstract

Realization of electrically pumped lasing with solution processable materials will have a revolutionary impact on many disciplines including photonics, chemical sensing, and medical diagnostics. Due to readily tunable, size-controlled emission wavelengths, colloidal semiconductor quantum dots (QDs) are attractive materials for attaining this goal. Here we use specially engineered QDs to demonstrate devices that operate as both a light emitting diode (LED) and an optically pumped laser. These structures feature a distributed feedback resonator integrated into a bottom LED electrode. By carefully engineering a refractive-index profile across the device, we are able to obtain good confinement of a waveguided mode within the QD medium, which allows for demonstrating low-threshold lasing even with an ultrathin (about three QD monolayers) active layer. These devices also exhibit strong electroluminescence (EL) under electrical pumping. The conducted studies suggest that the demonstrated dual-function (lasing/EL) structures represent a promising device platform for realizing colloidal QD laser diodes.

## Abstract (web summary)

Solution processable, electrically pumped lasers are a sought-after technology for many applications. Here the authors present dual-function devices based on colloidal quantum dots that behave as both electroluminescence structures and optically pumped lasers as a potential platform for electrically pumped quantum dot lasers.

## Introduction

Realization of lasers based on solution-processable materials has been a subject of much research across a range of fields including organic semiconductors^1–3^, perovskites^4–7^, and colloidal semiconductor nanomaterials^8–12^. In addition to simplifying fabrication protocols and reducing a device cost, a successful implementation of solution processable lasing devices would help expand the range of applications of lasing technologies into new areas including on-chip integrated photonic circuitry^13–15^, medical and biological imaging^16^, chemical sensing^17^, security^18^, and lab-on-a-chip diagnostics^19^. Among solution-processible light-emitting materials studied for lasing applications, colloidal nanocrystals of quantum dots (QDs) have gained considerable attention due to attractive characteristics such as a near-unity emission quantum yield^20^, a readily tunable emission wavelength^21,22^, and compatibility with various types of optical cavities. Despite these favorable features, realization of lasing with colloidal QDs is not straightforward. Because of a non-unity degeneracy of band-edge states, the condition for population inversion in a QD medium can only be met when the average number of electron-hole pairs (or excitons) per dot 〈*N*〉 is greater than one, implying that at least a fraction of dots in the sample must contain two or more excitons. Such multi-exciton states are subject to extremely fast nonradiative Auger decay whereby the electron-hole recombination energy dissipates in a form of a kinetic energy of the third carrier^23^. This process dominates over radiative recombination and leads to extremely fast (normally, sub-100 ps) deactivation of optical gain. This greatly complicates realization of lasing, especially, in the case of continuous wave (cw) optical or direct current (d.c.) electrical pumping.

Recent progress in the development of practical approaches for suppression of Auger decay (Auger-decay engineering)^11,24^ has produced several important advances in QD lasing research including the realization of cw lasing with optical pumping^10^, and more recently, demonstration of optical gain with d.c. electrical injection^25^. The next important milestone is the demonstration of a true lasing action with electrical pumping. Realization of this goal entails resolving several challenges including: (1) incorporation of a lasing cavity into an electroluminescent (EL) device without disrupting charge-carrier injection, (2) overcoming the problem of lasing suppression by conductive layers that are necessary elements of an EL structure, (3) realization of lasing with ultrathin QD active layers to enable electrical pumping, and (4) obtaining high current densities sufficient for achieving population inversion.

Here, we successfully resolve the first three of these challenges and demonstrate an operational EL device with a p-i-n light emitting diode (LED) architecture, which also functions as an optically pumped low-threshold laser. In these devices, we integrate a second-order distributed feedback (DFB) resonator into a cathode made of conductive indium tin oxide (ITO) whose refractive index is adjusted (lowered) such as to improve optical mode confinement within the active QD emitting layer (EML). By carefully designing the refractive index profile across the multilayered device stack, we are able to achieve a strong overlap between a planar waveguided mode and the QD active region, which is sufficient for demonstrating single-mode lasing with an ultrathin EML made of just three QD monolayers. Importantly, this strong lasing performance is realized in a complete LED device stack, which comprises multiple lossy conductive layers. Finally, we show that the fabricated structures also perform well as a standard p-i-n LED in EL measurements. The realized maximum current densities are still insufficient for inverting the population of the QD gain medium via electrical pumping. However, overcoming this remaining challenge should be possible by, for example, employing a current-focusing architecture^25^ and/or applying high-voltage pulsed bias^26,27^.

## Results

### Lasing with a DFB resonator integrated into a conductive ITO electrode

As we pointed out earlier, one challenge in realizing a functional colloidal QD laser diode (QLD) is the selection of an appropriate optical cavity design, which does not interfere with charge injection pathways and, at the same time, is not strongly susceptible to quenching of a gain medium by conductive electrodes and charge transport layers (CTLs). Previous research into QD lasing has explored various cavity designs including Fabry-Perot^28^, spherical^29^, and microring resonators^30^, vertical cavities implemented with distributed Bragg reflectors^31^, and DFB gratings^32^. Among those, a DFB resonator adjacent to a QD active region can, in principle, satisfy all requirements of a QLD architecture. In particular, it can be readily incorporated into an LED device stack without introducing any additional elements but by simply patterning one of the existing layers. Further, the cavity resonance can be readily adjusted by varying the grating period in the lateral device plane, without disturbing the device vertical structure, which can be thus used to independently tune carrier injection. In addition, a DFB structure is capable of producing high single-pass gain even with a thin optical-gain region as light amplification occurs in a lateral direction and hence the amplification length is not limited by the thickness of an active layer as in a vertical-cavity architecture. Finally, with the appropriate design of a cross-section profile of a refractive index across a device stack, it is possible to achieve good mode confinement within a thin QD EML and thereby reduce waveguiding losses arising from field penetration into adjacent conductive layers. Due to the above reasons, DFB resonators have been widely explored in past and ongoing efforts on realizing electrically pumped lasers based on organic materials^1,18,33^.

**Fig. 1:**
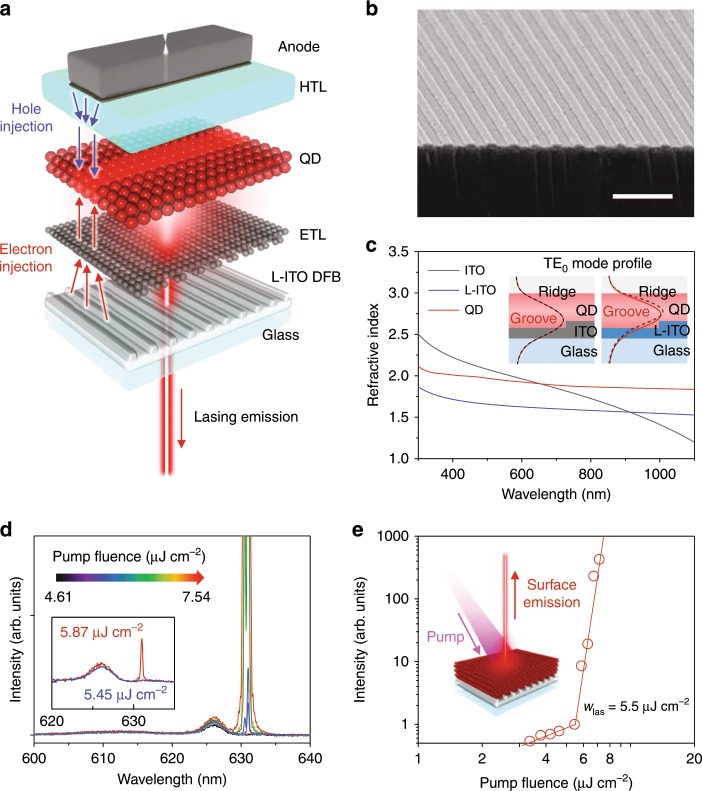
A QD laser with an L-ITO DFB cavity. **a** A proposed QLD architecture, which comprises a p-i-n multilayered QD-LED assembled on top of a DFB cavity integrated into a bottom L-ITO electrode. **b** A scanning electron microscopy (SEM) image of an L-ITO DFB grating with Λ = 370 nm. The scale bar is 1 µm. **c** Refractive indices of ITO, L-ITO and close-packed QDs as a function of wavelength. The inset shows calculated TE_0_ mode intensity profiles at λ = 630 nm across a device stack with a DFB resonator made of either standard ITO (left) or L-ITO (right). The red dashed and black solid lines show mode profiles for the groove and the ridge parts of the DFB grating, respectively. The greater difference between these profiles in the case of L-ITO compared to standard ITO indicates stronger optical feedback. **d** Surface emission spectra on the L-ITO-based device as a function of pump fluence displays a sharp transition to single-mode lasing (at λ = 630.9 nm) at *w* = 5.5 µJ cm^−2^; the inset shows emission spectra immediately before (blue) and immediately after (red) the lasing threshold. **e** Surface emission intensity versus pump fluence exhibits a well-pronounced threshold behavior with *w*_las_ = 5.5 µJ cm^−2^. The inset is a schematic of the L-ITO-based lasing device with a QD EML thickness of 200 nm. Source data are provided as a Source Data file.

In Fig. 1a, we display a possible structure of a DFB-based QLD. In the proposed design, the DFB resonator is embedded into an ITO cathode of an inverted QD LED wherein a thin QD EML is sandwiched between electron and hole transport layers (ETL and HTL, respectively) forming a p-i-n LED. To practically implement the proposed structure, we employ an optical gain layer based on continuously graded (cg) CdSe/Cd_*x*_Zn_1−*x*_Se/ZnSe_0.5_S_0.5_/ZnS QDs wherein the composition of a thick intermediate Cd_*x*_Zn_1−*x*_Se shell gradually varies from Cd-rich to Zn-rich with increasing distance from the CdSe core. As was demonstrated previously, these dots exhibit strongly reduced rates of nonradiative Auger recombination, which has been linked to the suppression of an intra-band transition involved in the dissipation of the energy released in the Auger decay event. The effectiveness of this approach for controlling Auger decay has been demonstrated by previous studies of cg-QDs conducted in the context of optically-excited lasing and electrically pumped optical gain^25^.

Compared to previous reports, the dots applied in the present study have been modified to further improve their lasing performance. Specifically, we reduce their overall size to increase the packing density and thereby boost optical gain. We increase the ZnSe_0.5_S_0.5_ shell thickness and further apply an external ZnS shell for improving chemical and photo-stability during device fabrication. The QDs used in our devices have a core diameter of 4 nm, the Cd_*x*_Zn_1−*x*_Se shell thickness of ca. 4 nm, and the ZnSe_0.5_S_0.5_/ZnS shell thickness of ca. 1.8 nm (see details of the QD synthesis in Methods). The QD emission is located at 618 nm and the emission quantum yield is ca. 80%. The radial compositional profile of the QDs, their transmission electron microscopy (TEM) images, and absorption and photoluminescence (PL) spectra are shown in Supplementary Fig. 1.

To test the ability of a DFB grating made of ITO to serve as an effective laser cavity, we first investigate structures in which QDs are directly deposited onto a patterned ITO substrate without additional layers used in LED devices. The DFB cavities are fabricated via laser interference lithography followed by dry etching as described in Methods and illustrated in Supplementary Fig. 2. The grating period (Λ) is selected so as to provide in-plane feedback via the 2nd-order Bragg diffraction, which corresponds to the condition λ_2B_ = *n*_eff_Λ, where λ_2B_ is the resonance wavelength, and *n*_eff_ is the effective refractive index. These structures also produce surface emission in a near-normal direction due to the 1st-order Bragg scattering, which is typically used to out-couple laser light (Fig. 1a). In order to match the 2nd-order Bragg resonance to the gain band at around 630 nm^25^, we prepare gratings with Λ from 360–380 nm (see an example of an SEM image in Fig. 1b). The cg-QDs are deposited via a multi-step spin-coating/cross-linking procedure (see Methods) to prepare a thick layer with thickness (*H*_QD_) of ca. 200 nm. The samples are excited by 130-fs, 3.1-eV frequency-doubled pulses of a femtosecond amplified laser using pump fluences (*w*) from ~1 µJ cm^−2^ to ~500 µJ cm^−2^ which correspond to average per-QD ‘excitonic’ occupancies (〈*N*〉) from ~0.2 to ~100 (see Methods).

We have started our experiments with a DFB grating incorporated into a layer of standard ITO. However, even with the highest pump fluence, these structures do not show a lasing effect. On the other hand, similar DFB structures made of SiO_2_ exhibit strong lasing performance with a low threshold (*w*_las_) of ~10 µJ cm^−2^ or 〈*N*_las_〉 ≈ 2 in terms of the excitonic QD occupancy (see Methods and Supplementary Fig. 3 for details of characterization of lasing performance). The detrimental effects of conductive electrodes on lasing have been pointed out in previous studies of devices based on organic semiconductors^34–36^, where they have been linked to direct quenching of emission from gain-active fluorophores and/or increased waveguiding losses due to optical-field penetration into conductive layers.

To test the possibility of direct quenching of QD emission by ITO, we investigate the effect of different electrodes (ITO and gold) on QD PL dynamics (Supplementary Fig. 4). For these measurements, we deposit QDs onto a pre-patterned ITO or a gold layer prepared on a glass substrate, and then compare the PL lifetime (τ_PL_) for each electrode with the corresponding reference value which is obtained for QDs deposited onto a bare glass side of the same substrate. In the case of a gold-coated substrate, which is a known emission quencher, PL decay of QDs is considerably shorter (by a factor of ~1.7) than that of the reference sample. On the other hand, the PL lifetime of QDs deposited onto a patterned-ITO layer (τ_PL_ = 7.1 ns) is nearly the same as for the reference sample (τ_PL_ = 7.7 ns). This suggests that the effect of emission quenching by ITO is insignificant and, therefore, the suppression of lasing in the case of the ITO grating is due primary to waveguiding losses arising from penetration of an optical mode into the conductive layer.

The modeling of light-intensity distribution within a QD/ITO/glass structure (Fig. 1c, left inset) does indicate that the field outside the QD layer is responsible for a large fraction (approximately half) of the total energy of the waveguided transverse electrical (TE_0_) mode. This is a direct consequence of a high refractive index of standard ITO (*n*_ITO_), which is comparable to that of the close-packed QDs (*n*_QD_ ≈ *n*_ITO_ ≈ 1.90 at λ = λ_2B_ = 630 nm, Fig. 1c) and, therefore, does not allow for good mode confinement within the QD active region.

To reduce the mode leakage outside the gain-active QD layer, we replace standard ITO with a mixture of ITO and SiO_2_ prepared by co-sputtering. Using this material, referred below to as ‘low-index ITO’ or L-ITO, we are able to reduce the refractive index of a patterned electrode to *n*_L-ITO_ = 1.6 (at λ = 630 nm; Fig. 1c), which leads to an appreciable improvement of field confinement within the QD EML (Fig. 1c, right inset). In addition, the increased contrast between the refractive indices of the DFB material and the QD gain medium leads to the enhanced coupling between counter-propagating in-plane waves, which improves optical feedback in the DFB resonator. The coupling coefficient κ can be calculated from κ = (|Δ*n*_eff_|/*n*_eff,ave_)Λ, where Δ*n*_eff_ is the difference between the effective refractive indices of the groove and the ridge parts of the DFB/QD structure, and *n*_eff,ave_ is the average of these quantities^37^. Based on our modeling (Fig. 1c, inset), κ is ~20 times higher in the case of the L-ITO DFB cavity compared to that made of standard ITO. This implies that the former structure offers considerably stronger optical feedback, which should facilitate lasing. Yet another benefit of L-ITO structures is virtually complete elimination of direct quenching of QD emission (which is weak but still discernible with standard ITO) as indicted by close similarity between PL decays for dots assembled on top of glass and L-ITO (Supplementary Fig. 4).

As a result of the above improvements, L-ITO DFB/QD devices behave as good single-mode lasers (Fig. 1d). In particular, they exhibit a very sharp lasing threshold above which the intensity of surface emitted light shows a very fast growth with the log-log slope of ~23 (Fig. 1e). The measured onset of the lasing effect is only ~5.5 µJ cm^−2^, which is comparable to the lowest values previously reported for any type of colloidal QD lasers^11^. The corresponding average per-dot excitonic occupancy, 〈*N*_las_〉, is 1.1 (see Methods section). This value is close to the theoretical gain threshold calculated for cg-QD in ref. ^38^ suggesting that the use of L-ITO as a DFB grating material indeed allows for realizing high-quality, low-loss optical cavities.

Near the threshold, lasing exhibits a single-mode behavior and is characterized by a narrow line at 630.9 nm with a resolution-limited linewidth of <0.2 nm (defined in terms of a full width at half maximum, FWHM). All of these observations are signatures of strong lasing performance, which is comparable to that of SiO_2_-DFB-based structures (Supplementary Fig. 3). Remarkably, it is realized with an optical cavity integrated directly into a conductive layer, which is capable of serving as an effective charge-injection electrode, as shown later in this work.

### Lasing in LED-like device stacks

**Fig. 2:**
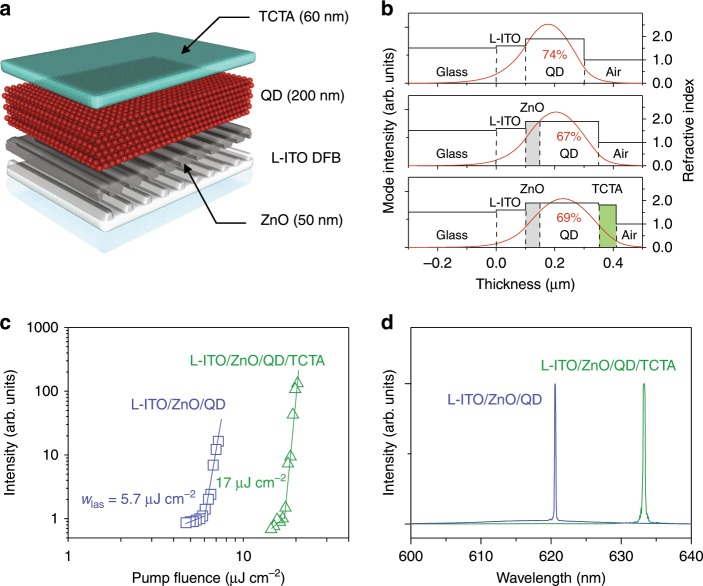
Effect of charge-transport layers adjacent to a QD active region on lasing. **a** Schematic representation of a multilayered QD DFB laser, which employs an LED-like p-i-n architecture with an inorganic (ZnO) ETL and an organic (TCTA) HTL. **b** Calculated TE_0_ waveguided mode intensity profiles (λ = 630 nm) for three different types of multilayered structures comprising an L-ITO DFB grating; the refractive indices of each layer are shown on the right vertical axes. Thanks to the low refractive index of L-ITO (*n*_L-ITO_ = 1.6) and a large thickness of the QD active region (*H*_QD_ = 200 nm), all structures exhibit strong mode confinement within the QD layer with the confinement factor Γ_QD_ = 67–74%. **c** Surface emission intensity as a function of pump fluence for two devices: one containing a ZnO ETL (*H*_ETL_ = 50 nm) inserted between the QDs and the DFB grating (blue), and the other containing both a ZnO ETL and a TCTA HTL (thicknesses 50 nm and 60 nm, respectively) on both sides of the QD active region (green); the lasing threshold for the two devices are 5.7 μJ cm^−2^ and 17 μJ cm^−2^, respectively. **d** Lasing spectra of the fabricated devices (color-matched to the data in **c**) exhibit a good single-mode behavior with lasing wavelengths of 620.6 nm (ZnO/QD structure) and 633.0 nm (ZnO/QD/TCTA structure). Source data are provided as a Source Data file.

Next, we investigate the effect of ETL and HTL on lasing performance using a device structure depicted in Fig. 2a. This device is a multilayered DFB laser, whose architecture is similar to that of a typical p-i-n QD-LED wherein a QD EML is placed between a tris(4-carbazoyl-9-ylphenyl)amine (TCTA) HTL and a ZnO ETL. The entire structure is assembled on top of an L-ITO DFB grating serving as an optical cavity. In Fig. 2b, we show simulated spatial profiles of a TE_0_ mode (at 630 nm) for a different number of layers in a device stack assuming that a QD layer has the same thickness (*H*_QD_ = 200 nm) as in our earlier lasing experiments. We use these simulations to calculate a mode confinement factor (Γ_QD_) defined as a ratio of an optical field energy confined within the QD active region to the total mode energy.

According to this modeling, in the case of a QD/L-ITO structure (no CTLs), Γ_QD_ = 74%. It decreases only slightly (to 67%) upon insertion of a ZnO interlayer but increases again (to 69%) after addition of a TCTA HTL. These calculations suggest that the introduction of CTLs is not supposed to significantly affect lasing performance of our devices. Indeed, the DFB laser with the ZnO ETL inserted between the QD layer and the L-ITO grating exhibits excellent lasing performance with a single-mode behavior and a low threshold of ~5.7 µJ cm^−2^ (Fig. 2c, d; blue data points). The device that comprises both a ZnO ETL and a TCTA HTL also lases but with a higher threshold of 17 µJ cm^−2^ (Fig. 2c, d; green data points). The increase in the lasing threshold is caused likely by the change of the effective refractive index upon deposition of TCTA leading to the shift of the DFB grating resonance away from the peak of the QD optical gain.

Our next step is to achieve lasing in devices wherein the thickness of a QD gain medium is reduced to values that would allow for effective electrical charge injection. Based on previous QD-LED research^39–43^, this thickness is about 1-to-3 QD monolayers, or ~50 nm or less for our 15.5-nm-diameter dots. In the case of such thin EMLs, a large fraction of waveguided light inevitably leaks into surrounding layers. The resulting decrease of the mode confinement factor reduces modal gain, which might complicate the realization of lasing and even completely suppress it. For example, in the case of a 50-nm QD layer in a QD-LED structure fabricated on top of L-ITO, Γ_QD_ drops to 23%, and it becomes only 9%, when the QD layer thickness is reduced to 20 nm (Supplementary Fig. 5). The drop in Γ_QD_ with decreasing *H*_QD_ is even more dramatic in the case of standard ITO with a higher reflective index, which tends to ‘pull’ the optical field away from the QD EML (Supplementary Fig. 6). This highlights once again the benefits of our L-ITO approach, which becomes especially important in the case of ultrathin QD active layers utilized in electrically pumped devices.

**Fig. 3:**
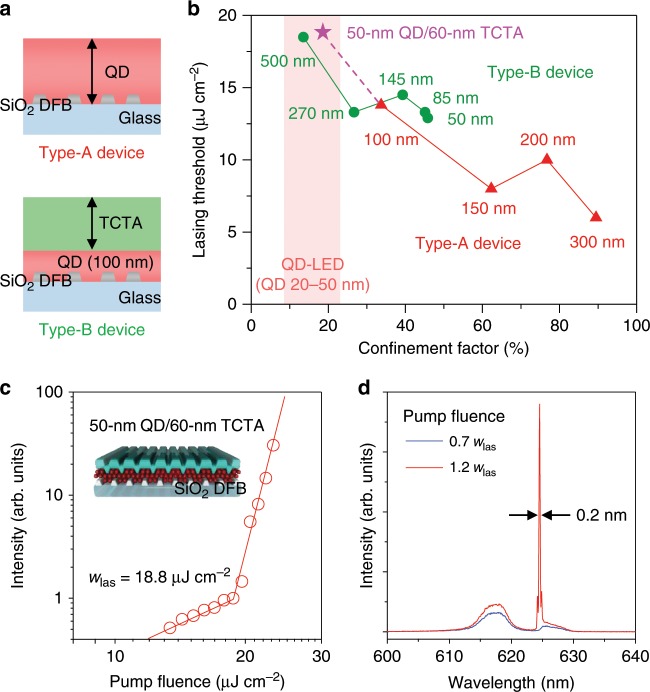
Effect of mode confinement factor on lasing. **a** In type-A (top) and type-B (bottom) devices, the mode confinement factor is tuned by varying, respectively, the thickness of the QD active region (*H*_QD_ = 50–300 nm) or the top TCTA layer (*H*_TCTA_ = 50–500 nm; *H*_QD_ is fixed at 100 nm). **b** Lasing threshold versus Γ_QD_ for type-A (red) and type-B (green) devices. A type-A device with *H*_QD_ = 50 nm does not lase due to the lack of a supported waveguided mode. However, lasing restores (magenta data point) upon addition of a 60-nm TCTA layer, which helps recover the device waveguiding properties. A type-A device with *H*_QD_ = 50 nm and an additional 60-nm TCTA layer and a type-B device with *H*_QD_ = 100 nm and *H*_TCTA_ = 500 nm have Γ_QD_ of 18.5% and 13.5%, respectively; these values fall within the range of Γ_QD_ of typical QD-LEDs (Supplementary Fig. 5). **c** A pump-intensity-dependent emission intensity of a type-A device with *H*_QD_ = 50 nm and *H*_TCTA_ = 60 nm (displayed in the inset). Despite an ultrathin QD gain region and a correspondingly low Γ_QD_ (=18.5%), the device exhibits a fairly low lasing threshold of 18.8 µJ cm^−2^. **d** The device shows a clear single-mode lasing behavior above the lasing threshold (*w* = 1.2*w*_las_ = 22.6 µJ cm^−2^) with a lasing wavelength of 624.6 nm. Source data are provided as a Source Data file.

To experimentally investigate the effect of a mode confinement factor on lasing, we fabricate two types of structures referred to as devices ‘A’ and ‘B’. A type-A device comprises a QD layer assembled on top of a SiO_2_ DFB grating (Fig. 3a, top), while a type-B device contains a QD EML and an additional top layer of TCTA (Fig. 3a, bottom). In the first type of devices, we modulate Γ_QD_ by changing the thickness of the QD layer from 50 nm to 300 nm. In the second type of devices, we keep *H*_QD_ constant (=100 nm) but change the HTL thickness (*H*_HTL_) from 50 nm to 500 nm. The optical confinement factors are calculated from the simulated TE_0_ mode intensity profiles (Supplementary Fig. 7). For each device, we optimize a grating period to account for changes in the effective refractive index and the resulting shift of a resonant wavelength.

The results of lasing measurements of fabricated structures are displayed in Fig. 3b, which shows lasing thresholds as a function of Γ_QD_ (lasing threshold behaviors are shown in Supplementary Fig. 7). For type-A devices, the mode confinement factor decreases from 89.5% to 33.7% when *H*_QD_ is reduced from 300 nm to 100 nm, which leads to the increase in the lasing thresholds from ~5 µJ cm^−2^ to ~13 µJ cm^−2^. The thinnest, 50-nm device does not show lasing up to the highest pump fluence applied in these measurements (~500 µJ cm^−2^). However, lasing in this case is suppressed not due to insufficient modal gain but because of the lack of a supported waveguided mode. In fact, we are able to achieve lasing in the same device after depositing 60 nm of TCTA on top of the QDs. The increase in the overall device thickness restores its waveguiding properties with Γ_QD_ of ~17%, and allows for observation of lasing with a fairly low threshold of 18.8 µJ cm^−2^ (Fig. 3c) and a clear single-mode lasing behavior (Fig. 3d). This observation is of great significance, as the QD layer thickness of 50 nm is typical of QD-LEDs and, therefore, it can be used for realizing electrically pumped devices.

For type-B devices, the mode confinement factor decreases as the thickness of the TCTA layer is increased, which leads to the expected increase in the lasing threshold. Importantly, a device with the thickest TCTA layer (*H*_TCTA_ = 500 nm) still shows good lasing performance with a fairly low threshold of ~18.5 µJ cm^−2^ despite an extremely small mode confinement factor of only 13.5%. This result confirms the assessment made based on type-A device measurements that the lasing action in our LED-like configuration is possible even with very low mode confinement factors that are comparable to those of typical high-performance QD-LEDs.

**Fig. 4:**
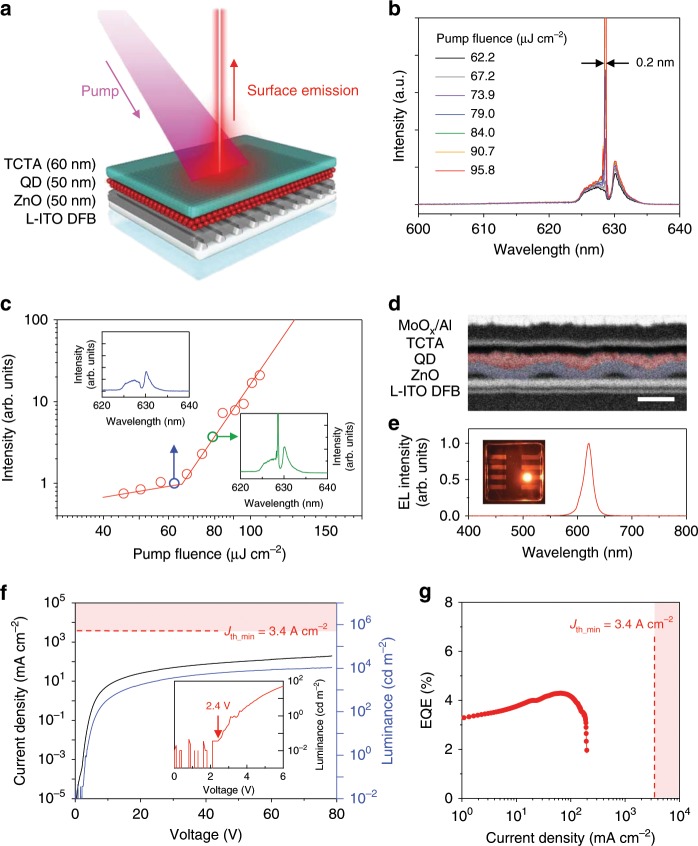
An LED-like device which performs as both an optically pumped single-mode laser and an electrically excited emitter. **a** Schematic illustration of an optically pumped multilayered DFB laser employing the same device architecture as a traditional p-i-n QD-LED. **b** Surface emission spectra on this device as a function of pump fluence display a clear transition to single-mode lasing at 629 nm. **c** Based on the onset of steep growth of the emitted light intensity, the lasing threshold is 65 µJ cm^−2^. Inset displays dramatic increase in emission intensity and line narrowing above the lasing threshold. **d** The cross-sectional SEM image of the EL device. To study EL, the device shown in **a** is supplemented by a hole-injecting electrode made of MoO_x_ (10 nm thickness) followed by Al (100 nm thickness). The scale bar is 200 nm. **e** The EL spectrum peaks at 621 nm. Inset: a photograph of an operating device, which shows that EL is highly uniform across the active area (0.15 × 0.15 cm^2^). **f** Current-density (luminance) versus voltage characteristics of the EL device; shaded area is the range of current densities where the QDs are expected to exhibit optical gain. Inset shows that the turn-on voltage of the EL device is ~2.4 V. **g** Device EQE as a function of current density indicates a peak EQE of 4.3%; the meaning of the shaded area is the same as in **f**. Source data are provided as a Source Data file.

As yet another test of feasibility of lasing in an LED-like structure with an ultrathin QD layer, we assemble on top of an L-ITO DFB grating a multilayered p-i-n device comprising a 50-nm QD layer placed between ZnO and TCTA CTLs (50 nm and 60 nm thicknesses, respectively) (Fig. 4a). The fabricated structure shows single-mode lasing at 628 nm (Fig. 4b) with a sharp threshold at 65.6 µJ cm^−2^ (Fig. 4c). The threshold is higher than for other laser systems discussed in this work. However, it is not due to reduced modal gain but is likely again due to a spectral shift of the DFB grating resonances as observed previously for structures with an added TCTA layer (Fig. 2c). In fact, based on the emission spectra, the lasing mode in our LED-like device develops on the higher-energy side of a photonic ‘stop band’, which is characterized by significant waveguiding losses due to enhanced surface emission^44^. This may explain the increase of the lasing threshold compared to other devices from this study, which operate on the lower-loss, low-energy side of the stop band (see, e.g., Fig. 3d).

### EL devices with a DFB-resonator-based charge-injection electrode

To evaluate the suitability of our L-ITO/DFB structures to function as electron-injecting electrodes in EL devices, we examine their charge transport properties. Using resistivity measurements (in both two-probe and four-probe configurations), we obtain that a typical sheet resistance (*R*_sh_) of the L-ITO/DFB grating is ~100 Ωsq^−1^. While being a factor of ~10 higher than for standard ITO (*R*_sh_ < 10 Ωsq^−1^; Supplementary Fig. 8), this value is comparable to (or even lower than) the sheet resistance of functional non-ITO transparent electrodes in literature devices including those based on graphene, silver nanowires, carbon nanotubes, and conducting polymers^45–47^. This suggests that our L-ITO/DFB structures should also act as fairly efficient charge-injection electrodes.

To realize an EL structure, we deposit a top MoO_x_/Al electrode onto the lasing multilayered stack from Fig. 4a. A cross-sectional SEM image of the fabricated device (Fig. 4d) shows a highly dense, thin QD layer (~3 monolayer thickness), which conformally covers a ZnO ETL without any apparent ‘pinholes’ and/or strong height modulation, which, if present, would result in spatially nonuniform injection. The biased structure indeed shows strong, spatially uniform EL at 621 nm confirming the absence of either ‘hot’ or ‘dead’ injection spots (Fig. 4e). The measured current density-voltage-luminance (*J*-*V*-*L*) characteristics in Fig. 4f show that the operating voltage is higher than those of typical QD-LEDs, which is a combined result of a higher resistivity of the L-ITO electrode compared to standard ITO and a fairly large thickness of the QD EML. While a 50-nm active medium is unusually thin by standards of QD lasing devices, it is still greater than typical thicknesses of emitting layers in QD LED structures.

Despite the high resistivity of the fabricated devices, their peak external quantum efficiency (EQE) is fairly good and reaches ~4.3%, which corresponds to the internal quantum efficiency of ~21.5%. Importantly, the EQE does not show a roll-off up to *J* = 0.1 A cm^−2^, indicating good balance between electron and hole injection currents (Fig. 4g). The maximum current density achieved in this device is 0.2 A cm^−2^, which is again limited by the high resistivity of the L-ITO electrode and the large thickness of the active QD layer.

## Discussion

Previously, optical gain in QD-LEDs has been demonstrated with currents >3.4 A cm^−2^ (ref. ^25^), which is around 20 times higher than the maximum current density realized in this study. This previous demonstration employed ‘current focusing’ achieved by reducing the injection area through the use of a tapered organic HTL. A similar ‘current-focusing’ design should also help increase current density in our LED-like lasing devices. Further, the use of high voltage pulsed excitation can be utilized as a supplementary tool for boosting the driving current^26,27^.

An additional challenge is the need for an alternative top contact. While the use of the MoO_x_/Al electrode allows us to achieve a fairly good EL performance, it leads to suppression of lasing, which is a known effect of metal electrodes^48–50^. Even though such electrodes may not affect EL, they have a considerably greater effect on lasing as optical amplification requires multiple passes of the emitted light through the gain medium, which leads to accumulation of plasmonic losses. A possible solution to this problem is the use of a non-metal top contact such as graphene^51^ or metal oxide^52^. As was shown previously, both of these materials exhibit significantly reduced plasmonic losses compared to conventional metal contacts^53,54^.

To summarize, we have developed an LED-like multilayered DFB laser, which utilizes cg-QDs with suppressed Auger decay as a gain medium and a second-order DFB grating integrated into an L-ITO electrode as an optical cavity. We demonstrate that the proposed design represents a highly robust lasing platform, which is tolerant of a wide variation of device structural parameters (e.g., type, number, and thickness of device layers) and is capable of low-threshold lasing even with thin (~50 nm) QD optical-gain layers. We further show that these lasing structures can also operate as electrically pumped LEDs with a good EQE ‘roll-off’ behavior. Together with the previous work on electrically excited QD optical gain^25^, the conducted study proves the feasibility of colloidal QD lasing with electrical injection and further demonstrates a practical architecture for implementing a QLD device.

## Methods

### Synthesis of cg-QDs

To synthesize cg-QDs with a CdSe/Cd_*x*_Zn_1−*x*_Se/ZnSe_0.5_S_0.5_/ZnS structure, we applied a modified version of the synthetic procedure from ref. ^25^. All reactions were carried out under inert atmosphere. Briefly, CdSe wurtzite cores with the 2 nm radius, 1 mL of n-trioctylphosphine (TOP), and 4 mL of 1-octadecene were placed into a reaction vessel and the temperature was quickly raised to 310 °C. To grow a compositionally-graded Cd_*x*_Zn_1−*x*_Se shell on top of the CdSe cores, 2 mL of 0.5 M zinc oleate [Zn(OA)_2_] were added to the mixture and the solution containing 3 mL of 2 M TOPSe (selenium dissolved in TOP), 3 mL of 0.5 M cadmium oleate, and 6 mL of 1-octadecene was continuously fed into the reaction at a constant rate of 5 mL h^**−**1^. Two microliter of Zn(OA)_2_ were added into the reactor every 40 min. The reaction duration required to prepare CdSe/Cd_*x*_Zn_1−*x*_Se QDs with the overall radius of 6 nm was ~120 min. In the following step, a high-band-gap ZnSe_0.5_S_0.5_ shell was deposited on top of the CdSe/Cd_x_Zn_**1−*x***_Se QDs by injecting 8 mL of 0.5 M Zn(OA)_2_ at once and slowly adding a mixture containing 1 mL of 2 M TOPS, and 1 mL of 2 M TOPSe using the injection rate of 4 mL h^**−**1^. In order to prepare an additional ZnS protective layer, we applied the same procedure as one used for the ZnSe_0.5_S_0.5_ shell growth; in this procedure, we used a mixture of 2 mL of 0.5 M Zn(OA)_2_ and 0.5 mL of 2 M of TOPS. The reaction products were purified by a five-step precipitation/redispersion method with toluene and acetonitrile. The purified QDs were dispersed in octane and used as needed.

### Fabrication of DFB optical cavities

Second-order DFB optical cavities were fabricated via laser interferometric lithography. Low refractive index ITO (L-ITO) or standard ITO substrates were purchased from Thin Film Devices Inc. An anti-reflection coating (ARC) (i-CON 16, Brewer Science, Inc.) and a negative tone photoresist (PR) (NR7-500P, Futurrex Inc.) were sequentially spin coated onto the L-ITO substrates and baked. For patterning PR, we used a third harmonic output of a ND:YAG laser (λ = 355 nm), and a Lloyd’s mirror setup was applied to create an interference pattern used to define a one-dimensional grating. The grating period (Λ) was controlled by changing the angle between the incident and the reflected beams (θ) according to equation Λ = λ/2sinθ. The exposed samples were post-baked at 100 °C for 1 min and then developed in a developer (RD6, Futurrex Inc.) to imprint a one-dimensional periodic structure onto a PR layer (Supplementary Fig. 2). Using the patterned PR layer as an etching mask, inductively coupled plasma reactive ion etching (ICP-RIE) was performed to etch ARC by using O_2_, and then ITO was sequentially etched by the Cl_2_/O_2_ mixture gas (the flow rates of Cl_2_ and O_2_ were 60 sccm and 5 sccm, respectively; the chamber pressure was 5 mTorr). Finally, PR and ARC were stripped away using a high-power O_2_ plasma, which resulted in a DFB grating imprinted onto the ITO layer. To prepare SiO_2_ DFB structures, a 40-nm layer of SiO_2_ was deposited using an electron-gun evaporator. This was followed by the same procedures as those used to fabricate ITO DFB gratings, except that the unmasked SiO_2_ area was etched away using a gaseous mixture of CHF_3_ and O_2_ (the flow rates of CHF_3_ and O_2_ were 60 sccm and 5 sccm, respectively; the chamber pressure was 5 mTorr).

The grating period of DFB resonators was selected to provide in-plane feedback according to the relationship for the 2nd-order Bragg diffraction, λ_2B_ = *n*_eff_Λ. The fabricated gratings had Λ of 360–380 nm and 350–400 nm for L-ITO and SiO_2_, respectively. The grating groove height was 40 nm, which allowed for strong in-plane feedback, and at the same time, did not lead to any appreciable distortion of a multilayer QD-LED structure composed of thin films with thicknesses of several tens of nm. The duty cycle (i.e., the ratio of the groove width and the grating period) was ~0.3, which was chosen based on the requirement to ensure strong coupling between counter-propagating in-plane waves^55^.

### Fabrication of QD DFB lasers

QD films were deposited on top of DFB gratings using a multi-step coating/crosslinking procedure, which produced highly robust, spatially uniform, optically clear films with a wide range of thicknesses. 1,8-diaminooctane was used as a linking agent. During each coating cycle, spin casting of a 50 mg mL^−1^ QD solution was followed by drop-wise application of a 2.85-mg/mL 1,8-diaminooctane solution in methanol and then by a 1 min wait time to ensure the completion of a cross-linking reaction. After that, the film was rinsed with methanol to remove unreacted linking groups. One cycle produced a QD film of a ca. 50-nm thickness. To prepare a thicker QD layer, the coating/cross-linking cycle was repeated a required number of times.

Fabricated devices usually showed lasing into a transverse electrical (TE) mode (see Supplementary Fig. 3d, e). The effective refractive index of the QD films (*n*_eff,TE_) was determined from the expression for the 2nd-order Bragg scattering using the known DFB grating period and the measured position of the center of the photonic gap observed for the TE polarization. Based on the measurements of 17 devices, the average value of *n*_eff,TE_ was 1.76. A device-to-device variation in *n*_eff,TE_ was within ±0.05 (Supplementary Fig. 3f). Despite a fairly small uncertainty in the refractive index (<3%), the resulting variation in λ_2B_ was quite large (±18 nm). Therefore, for each type of a multilayered device, we fabricated several gratings with different periods (see previous section) and upon completion of a device stack, we characterized their Bragg patterns. For lasing experiments, we selected samples which showed the closest match between the Bragg TE mode and the optical gain band. In the case of devices with especially good spectral matching, we were able to achieve lasing thresholds which were near the fundamental limit of one electron-hole pair per dot on average (see, e.g., Fig. 1e).

### DFB lasing measurements

In lasing experiments, cg-QD/DFB devices were excited using a 400-nm second-harmonic output of a Spectra Physics Spitfire amplifier, which produced ~130-fs, 1 kHz pulses. The pump beam was focused with a cylindrical lens into a spot with the 30 × 390 µm^2^ dimensions. The spectrally dispersed surface emitted light was detected with a liquid-nitrogen-cooled charge-coupled device of a SpectraPro-500i apparatus (spectral resolution of ~0.1 nm). The average per-QD ‘excitonic’ occupancy 〈*N*〉 was calculated from 〈*N*〉 = σ(*w*/*hv*_p_), where σ is the QD absorption cross-section, *w* is the per-pulse pump fluence, and *hv*_p_ (=3.1 eV) is the pump photon energy. For the studied QD samples, σ at 3.1 eV is 1.0 × 10^−13^ cm^2^, as determined from saturation of a long-time signal in pump-intensity-dependent PL dynamics^38^. All measurements were performed at room temperature. A summary of lasing thresholds observed for studied devices is provided in Supplementary Table 1.

Below the threshold pump fluence (*w*_las_), we observed narrow (~3 nm full width at half maximum, FWHM), linearly-polarized transverse electrical (TE) and transverse magnetic (TM) cavity modes along with a weaker, unpolarized, spectrally broad (~20 nm FWHM) emission. When the pump fluence was raised above *w*_las_, a sharp spectral line (~0.2 nm FWHM) emerged on top of one of the cavity modes (typically, a lower-energy TE peak). The intensity of this line exhibited very fast, super-linear growth with increasing pump level characterized by a log–log slope of 10–20 or more. The outcoupled light was highly directional and propagated in the direction normal to the DFB surface, as expected for the second-order DFB cavity (Supplementary Fig. 3b, c). All of these observations were consistent with the lasing action in a QD/DFB cavity and could not be explained by alternative processes such as amplified spontaneous emission, directional scattering, or cavity modified fluorescence. The first of these effects would not be linked to cavity resonances, while the other two would not exhibit a well-defined threshold behavior.

### Waveguided mode calculations

The mode intensity profiles and effective refractive indices for the studied multilayered structures were calculated using the “1-D Waveguided Mode Solver for Dielectric Multilayer Slab Waveguides”^56^. The confinement factors were calculated as the ratio of an optical field energy confined within a specific layer to the total mode energy. The experimental values of refractive indices were obtained using a rotating-compensator ellipsometer with a diode detector array and a xenon light source (J.A. Woollam Co. M-2000).

### LED fabrication and characterization

A ZnO ETL was fabricated using a sol–gel process^57^. Briefly, 1 g of zinc acetate dehydrate (Zn(CH_3_COO)_2_∙2H_2_O) was dissolved in 10 mL of 2-methoxyethanol (CH_3_OCH_2_CH_2_OH), which was followed by addition of 0.28 g of ethanolamine. The ZnO precursor solution was stirred overnight for ~15 h before use. A spin-coated ZnO film was annealed at 200 °C for 2 h in ambient air. TCTA, MoO_x_, and Al were thermally evaporated with a shadow mask under high-vacuum condition (0.5–1 × 10^**−**6^ Torr). The active device area was 0.0225 cm^2^ (0.15 × 0.15 cm^2^). The current density-luminance-voltage (*J*-*L*-*V*) characteristics were measured using a source-measurement unit (Keithley 237) and a calibrated Si photodiode coupled to an optical power meter (Newport 1830C). EL spectra were obtained using an Ocean Optics USB2000 spectrometer, and luminance of devices was calibrated using a spectroradiometer Konica-Minolta CS-1000.

### Reporting summary

Further information on research design is available in the Nature Research Reporting Summary linked to this article.

## Supplementary information

Supplementary Information

Peer Review File

Reporting Summary

Source Data File

## Data Availability

The data that support the findings of this study are available from the corresponding author upon reasonable request. The source data underlying Figs. 1c–e, 2b, 3b–d, 4b, c, e–g and Supplementary Figs. 1c, 3c–f, 4, 5, 6, 7, and 8 are provided as a Source Data file.
