# Supplementary material for: Optically pumped colloidal-quantum-dot lasing in LED-like devices with an integrated optical cavity
Source: Nat Commun. 2020 Jan 14;11:271. doi: 10.1038/s41467-019-14014-3 (PMC6959307; doi:10.1038/s41467-019-14014-3)
Supplement: Supplementary file 1 — Supplementary Information [file 41467_2019_14014_MOESM1_ESM.pdf]

Optically Pumped Colloidal-Quantum-Dot Lasing in LED-Like Devices with an Integrated Optical Cavity

Roh et al.

Supplementary Information

Optically Pumped Colloidal-Quantum-Dot Lasing in LED-Like Devices with an Integrated Optical Cavity

Jeongkyun Roh[1,†,‡], Young-Shin Park[1,2†,], Jaehoon Lim[1,3], and Victor I. Klimov[1*]

[1]Chemistry Division, Los Alamos National Laboratory, Los Alamos, New Mexico 87545, USA

[2]Centre for High Technology Materials, University of New Mexico, Albuquerque, New Mexico, 87131, USA

[3]Department of Chemical Engineering & Department of Energy System Research, Ajou University, Suwon 16499, Republic of Korea

[†]These authors contributed equally to this work
[‡]Present address: Department of Electrical Engineering, Pusan National University, Busan 46241, Republic of Korea

[*]Address correspondence to klimov@lanl.gov

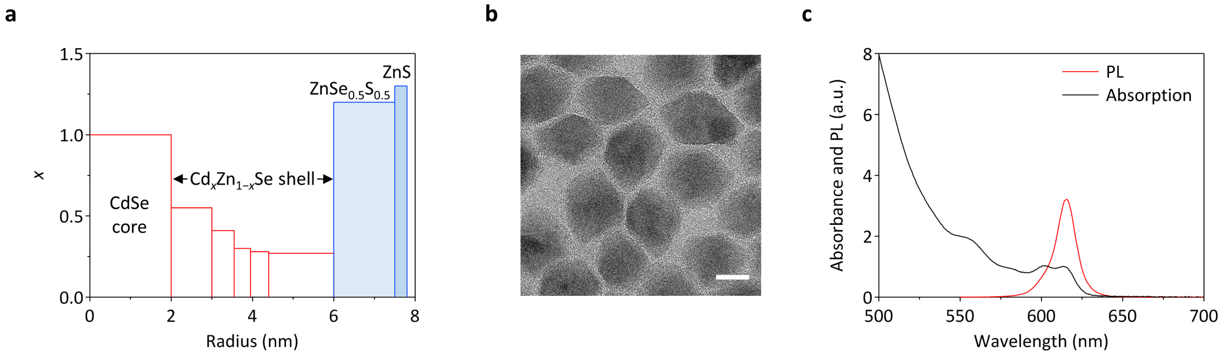

Supplementary Fig. 1| a, The radial compositional profile of continuously graded (cg) CdSe/ $Cd_xZn_{1-x}Se/ZnSe_{0.5}S_{0.5}$ /ZnS quantum dots (QDs) used in this study. The CdSe core has a radius of 2 nm. The composition of the intermediate $Cd_xZn_{1-x}Se$ shell gradually varies from Cd-rich to Zn-rich with increasing distance from the CdSe core. The final layers of $ZnSe_{0.5}S_{0.5}$ (~1.5-nm thickness) and ZnS (~0.3 nm thickness) are grown to improve chemical and photo-stability. **b,** transmission electron microscopy (TEM) image of the cg-QDs showing the average diameter of 15.5 nm. The scale bar is 10 nm. **c,** Absorption (black) and photoluminescence (PL, red) spectra of the cg-QDs used in this study. The PL peak is located at 618 nm and the emission quantum yield is ca. 80%. Source data are provided as a Source Data file.

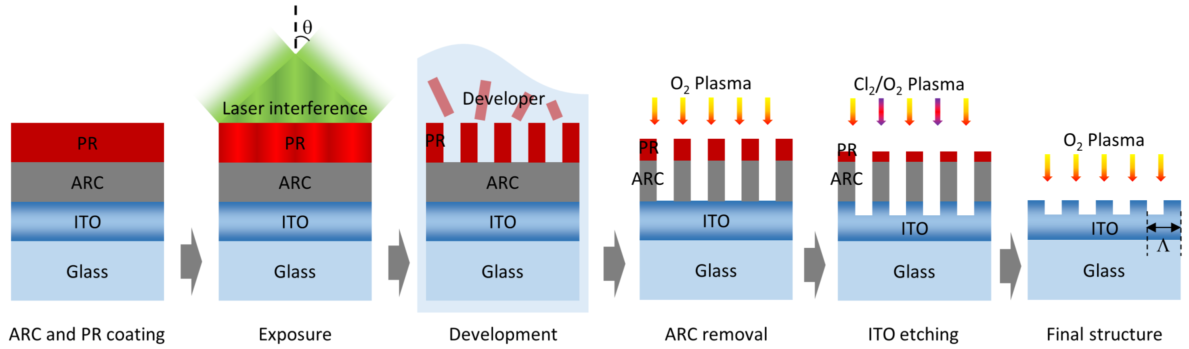

Supplementary Fig. 2| Fabrication of indium tin oxide (ITO) distributed feedback (DFB) cavities using a laser interference lithography. An anti-reflection coating (ARC) and a photoresist (PR) were spin coated on pre-patterned ITO substrates, and then a PR layer was periodically patterned using laser interference, which was followed by the development. Using the patterned PR layer as an etching mask, ARC was removed by an O_2 plasma and then ITO was partially etched by a Cl_2/O_2 plasma. Finally, PR and ARC were etched away by a high-power O_2 plasma.

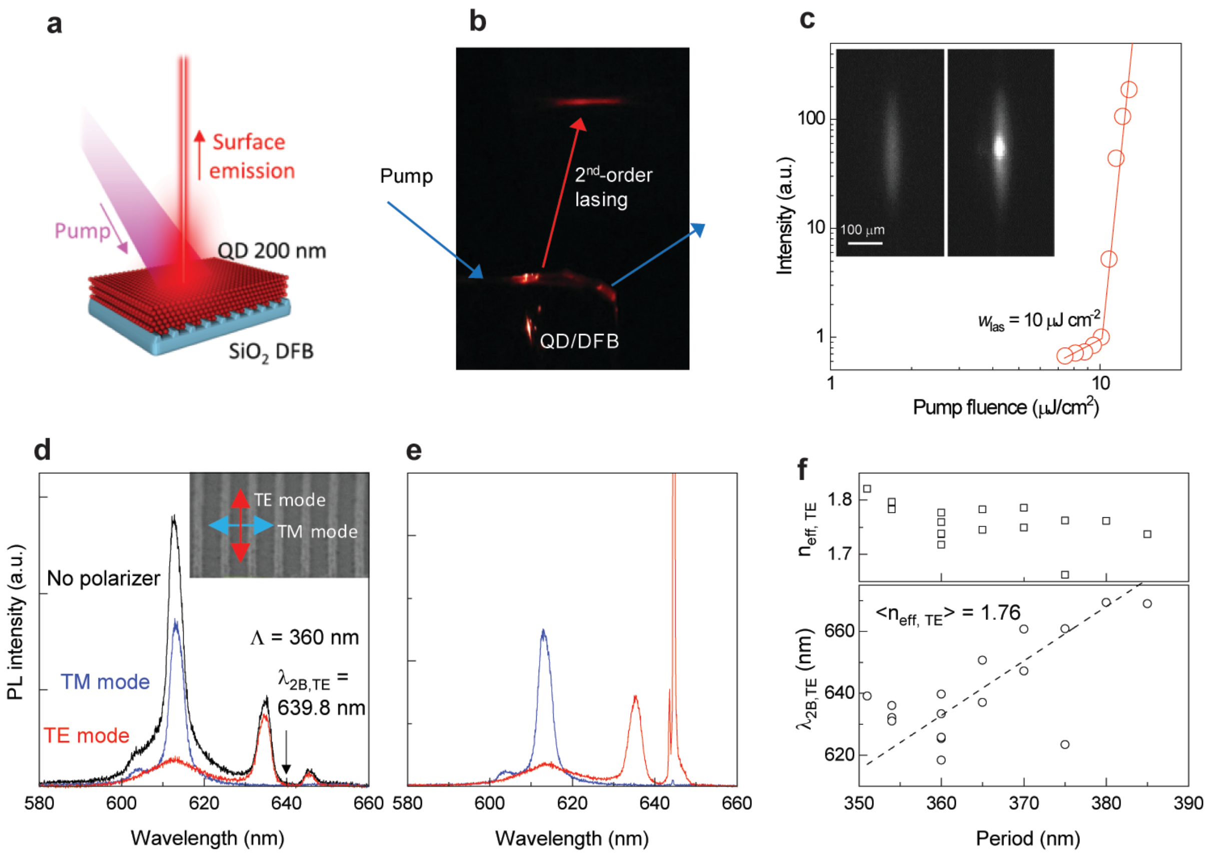

Supplementary Fig. 3| a, A schematic depiction of a QD laser based on the second-order SiO_2 DFB resonator. **b,** A photograph of an operating device which lases in the direction normal to the DFB surface, as expected for the second-order grating. **c,** Surface emission intensity as a function of pump fluence indicates a sharp lasing threshold (w_{las}) of ~10 μJ cm^{-2}. The insets show spatial profiles of a stripe-shaped pump beam (~30×390 μm^2 area) focused onto a QD/DFB device before (left) and after (right) the lasing threshold. The bright spot in the middle of the excited device area is lasing emission in the direction normal to the DFB grating due to out-of-plane 1st-order scattering. **d,** Emission spectra of the QD/DFB cavity before the lasing threshold measured without a polarizer (black) and with a linear polarizer for polarizations along (TE mode; the red arrow in the inset and the red trace in the main panel) and perpendicular (TM mode; the blue arrow in the inset and the blue trace in the main panel) to the DFB grating grooves. **e,** Polarization-resolved measurements above the lasing threshold (same color coding as in 'd') indicate that lasing occurs into the linear-polarized TE mode. **f,** The wavelength of the 2nd-order TE Bragg resonance, $\lambda_{2B,TE}$ (based on the position of the center of the TE stop band; panel 'd'), as a function of grating period, Λ (bottom sub-panel). The effective refractive index ($n_{eff,TE}$) determined from the Bragg condition based on the measured $\lambda_{2B,TE}$ as a function of Λ (top sub-panel). Source data are included as the Source Data file.

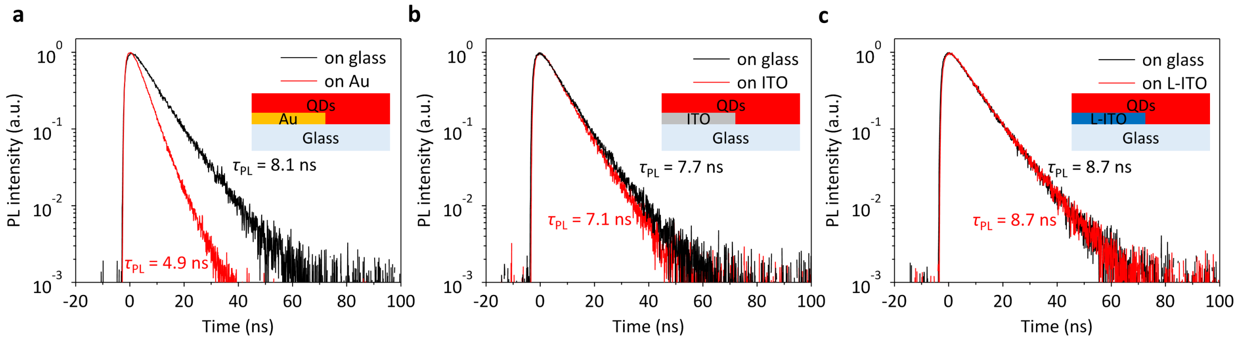

Supplementary Fig. 4| a-c, PL decays of QD films (50 nm thickness) on Au (**a**), standard ITO (**b**), and low refractive index ITO (L-ITO) (**c**) (red traces); the PL decays of QDs assembled on the bare glass side of the same substrates are used as a reference value for each sample (black traces). The PL decay of QDs on Au is considerably faster (PL lifetime, τ_{PL} = 4.9 ns) than that of the reference sample (τ_{PL} = 8.1 ns). On the other hand, τ_{PL} is just slightly shorter for standard ITO (τ_{PL} = 7.1 ns) than for the reference lifetime (τ_{PL} = 7.1 ns) and is virtually the same as for the reference sample for L-ITO (τ_{PL} = 8.7 ns). Source data are provided as a Source Data file.

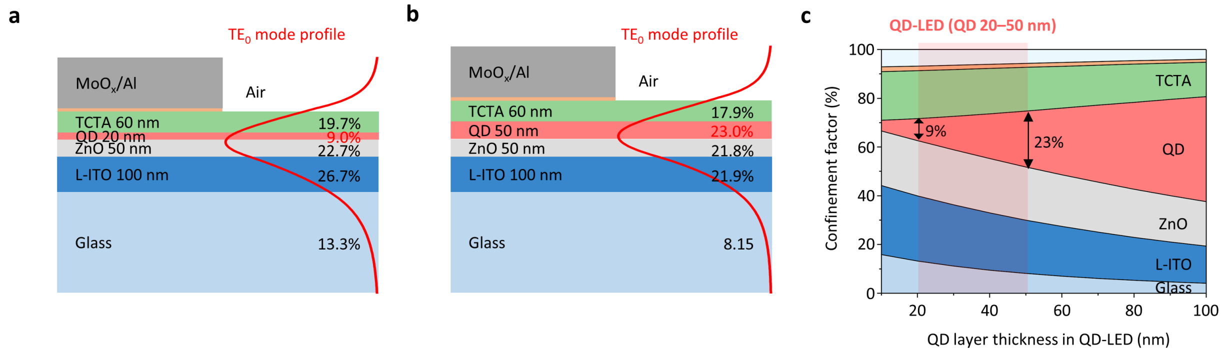

Supplementary Fig. 5| a–b, The TE_0 waveguide mode profiles in QD-LEDs with a bottom electrode based on L-ITO. The mode distributions for LEDs with a QD layer thickness of 20 nm (**a**) and 50 nm (**b**). **c,** The TE_0 mode confinement factors for each layer of a QD-LED as a function of thickness of the QD active region. Source data are provided as a Source Data file.

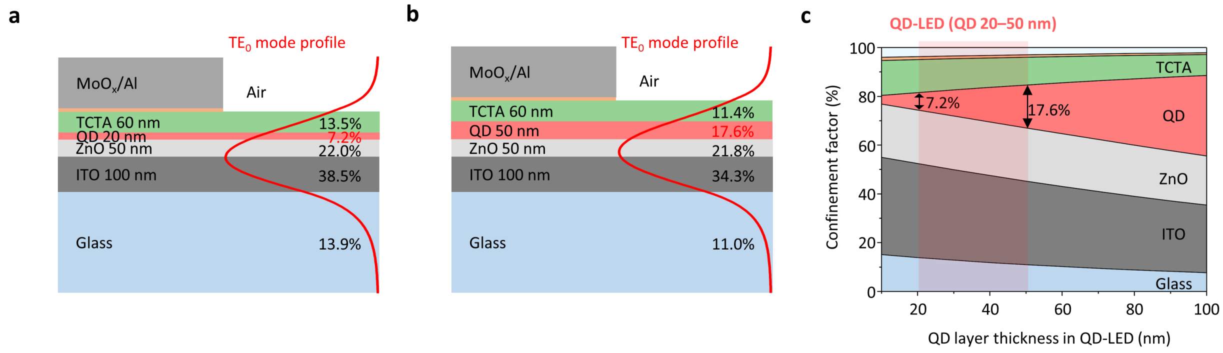

Supplementary Fig. 6| Same as in Supplementary Fig. 5 but for devices with a bottom electrode based on standard ITO. Source data are provided as a Source Data file.

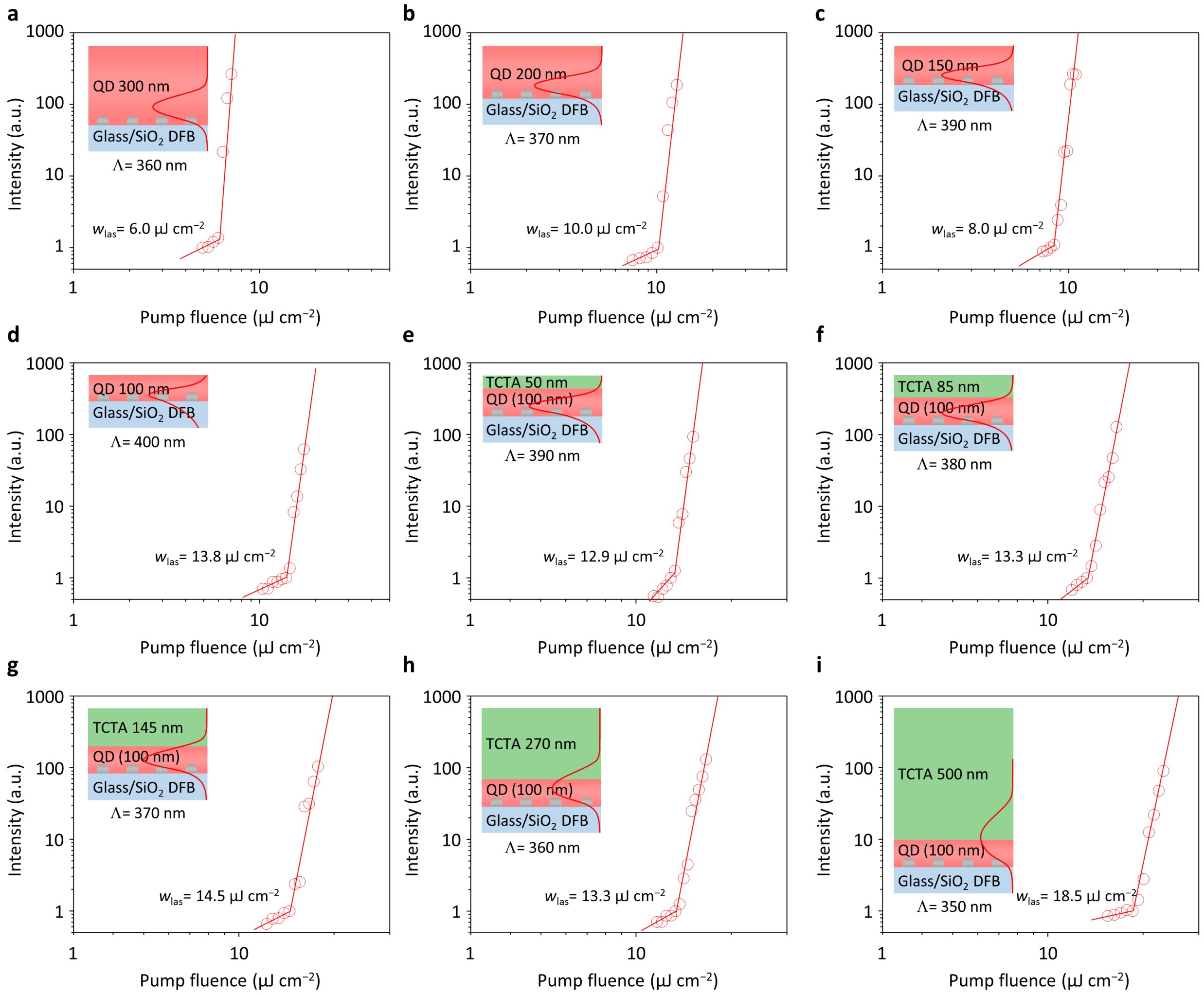

Supplementary Fig. 7| a–d Lasing threshold behaviors of a type-A device with different thicknesses of the QD active region (H_{QD}); H_{QD} = 300 nm (**a**), 200 nm (**b**) (same data as in Supplementary Fig. 3b), 150 nm (**c**), and 100 nm (**d**). **e–i** Lasing threshold behaviors of a type-B device with H_{QD} = 100 nm and different thicknesses of the TCTA layer (H_{TCTA}); H_{TCTA} = 50 nm (**e**), 85 nm (**f**), 145 nm (**g**), 270 nm (**h**), and 500 nm (**i**). Insets show a device structure and a corresponding TE_0 mode profile. Source data are provided as a Source Data file.

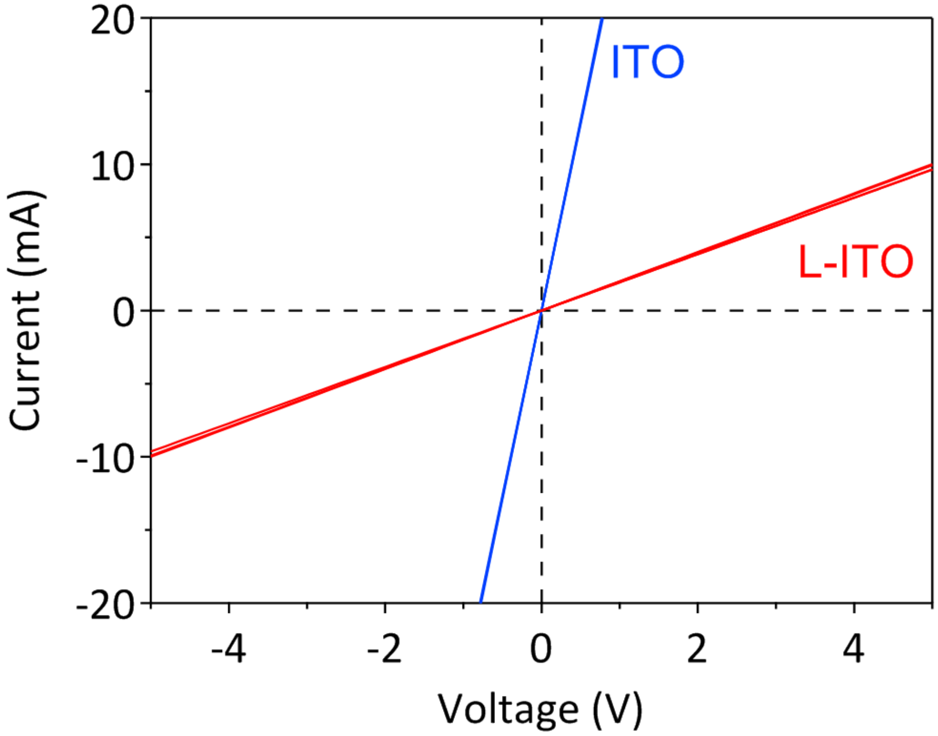

Supplementary Fig. 8| Two-point probe resistivity measurements of ITO and L-ITO. Source data are provided as a Source Data file.

DFB material	Device structure	Lasing threshold (μJ cm^{-2})	Figure
SiO_2	DFB/QD100	13.8	3b
	DFB/QD100	24.3	
	DFB/QD150	8.0	3b
	DFB/QD200	10	3b
	DFB/QD200	14.5	
	DFB/QD200	21	
	DFB/QD300	6.0	3b
	DFB/QD50/TCTA60	18.8	3c
	DFB/QD100/TCTA50	12.9	3b
	DFB/QD100/TCTA85	13.3	3b
	DFB/QD100/TCTA145	14.5	3b
	DFB/QD100/TCTA270	13.3	3b
	DFB/QD100/TCTA500	18.5	3b
	DFB/QD150/TCTA60	11.5	
	DFB/QD150/TCTA60	19.6	
	DFB/QD200/TCTA60	24.5	
	DFB/QD200/TCTA60	28	
	DFB/QD200/TCTA60	30.8	
L-ITO	DFB/QD200	13	
	DFB/QD200	15	
	DFB/QD250	5.5	1e
	DFB/QD250	18	
	DFB/QD200/TCTA60	17	
	DFB/ZnO50/QD200	8.1	
	DFB/ZnO50/QD250	8.5	
	DFB/ZnO50/QD250	5.7	2c
	DFB/ZnO50/QD250/TCTA60	17	2c
	DFB/ZnO50/QD50/TCTA60	65	4c

Supplementary Table 1| The summary of lasing thresholds observed for 28 devices fabricated and tested in the present study. The number, which appears in the 2nd column next to the type of the specific device layer (QD, ZnO, or TCTA) indicates its thickness in units of nm. During a given test, the fabricated devices showed stable performance on a time scale of over 10 hours of continuous operation. This performance was reproducible for several days.
