# Supplementary material for: Optically pumped colloidal-quantum-dot lasing in LED-like devices with an integrated optical cavity
Source: Nat Commun. 2020 Jan 14;11:271. doi: 10.1038/s41467-019-14014-3 (PMC6959307; doi:10.1038/s41467-019-14014-3)
Supplement: Supplementary file 3 — Reporting Summary [file 41467_2019_14014_MOESM3_ESM.pdf]

nature research

Corresponding author(s): Victor I. Klimov

Lasing Reporting Summary

Nature Research wishes to improve the reproducibility of the work that we publish. This form is intended for publication with all accepted papers reporting claims of lasing and provides structure for consistency and transparency in reporting. Some list items might not apply to an individual manuscript, but all fields must be completed for clarity.

For further information on Nature Research policies, including our data availability policy, see Authors & Referees.

Experimental design

Please check: are the following details reported in the manuscript?

1. Threshold

Plots of device output power versus pump power over a wide range of values indicating a clear threshold

☒ Yes ☐ No

Fig. 1e, Fig. 2c, Fig. 3c, and Fig.4c.

2. Linewidth narrowing

Plots of spectral power density for the emission at pump powers below, around, and above the lasing threshold, indicating a clear linewidth narrowing at threshold

☒ Yes ☐ No

The inset of Fig. 1d, Fig. 3d, and Fig. 4b.

Resolution of the spectrometer used to make spectral measurements

☒ Yes ☐ No

0.1 nm; see subsection "DFB lasing measurements" in Methods of the revised manuscript.

3. Coherent emission

Measurements of the coherence and/or polarization of the emission

☒ Yes ☐ No

Supplementary Fig. 3d and 3e in the revised manuscript.

4. Beam spatial profile

Image and/or measurement of the spatial shape and profile of the emission, showing a well-defined beam above threshold

☒ Yes ☐ No

Supplementary Fig. 3b of the revised manuscript.

5. Operating conditions

Description of the laser and pumping conditions
Continuous-wave, pulsed, temperature of operation

☒ Yes ☐ No

See subsection "DFB lasing measurements" in Methods of the revised manuscript.

Threshold values provided as density values (e.g. W cm^{-2} or J cm^{-2}) taking into account the area of the device

☒ Yes ☐ No

Fig. 1e, Fig. 2c, Fig.3c, and Fig. 4c

6. Alternative explanations

Reasoning as to why alternative explanations have been ruled out as responsible for the emission characteristics
e.g. amplified spontaneous, directional scattering; modification of fluorescence spectrum by the cavity

☒ Yes ☐ No

See subsection "DFB lasing measurements" in Methods of the revised manuscript.

7. Theoretical analysis

Theoretical analysis that ensures that the experimental values measured are realistic and reasonable
e.g. laser threshold, linewidth, cavity gain-loss, efficiency

☒ Yes ☐ No

Lines 6-11 on page 8 of the revised manuscript..

8. Statistics

Number of devices fabricated and tested

☒ Yes ☐ No

28; see test results in Supplementary Table 1 of the revised manuscript.

Statistical analysis of the device performance and lifetime (time to failure)

☒ Yes ☐ No

Supplementary Table 1 of the revised manuscript.

March 2018
