# Supplementary material for: Optically pumped colloidal-quantum-dot lasing in LED-like devices with an integrated optical cavity
Source: Nat Commun. 2020 Jan 14;11:271. doi: 10.1038/s41467-019-14014-3 (PMC6959307; doi:10.1038/s41467-019-14014-3)
Supplement: Supplementary file 2 — Peer Review File [file 41467_2019_14014_MOESM2_ESM.pdf]

Reviewers' comments:

Reviewer #1 (Remarks to the Author):

The manuscript titled “Optically Pumped Colloidal-Quantum-Dot Lasing in LED-Like Devices with an Integrated Optical Cavity” reports the demonstration of the lasing performance of ultrathin devices made of CdSe based colloidal quantum dots (QD) incorporated into LED configurations with the conductive ITO electrode serving as the grating plane for a DFB laser cavity. The authors start with discussion of challenges needed to be overcome to achieve true electrically pumped lasing from colloidal quantum dots, then demonstrate a continuously graded (cg) QDs with the DFB resonator embedded into an ITO cathode of an inverted QD LED and explain how this specific device could fulfill the requirements to solve the above challenges. From simulation and experimental results, they claim that LED-like device could behave as good single mode lasers with reduced mode leakage. Later, the authors achieved EL with fairly good EQE based on the same LED-like device with DFB resonator. Although this work did not realize electrically pumped lasing from colloidal quantum dots, there are substantial advances in important aspects underlying electrically pumped colloidal lasers, such as cavity platform, effects of electrodes on gain medium, electrical injection etc. Over all, this work will help researchers to understand the key factors for achieving electrically-pumped lasing and will inspire them to fabricate new photonic devices. Therefore, I suggest to accept this manuscript if the authors are able to address the following issues:

1). In the first part of result section, the author proposed to use the similar dots applied in previous reports but with reduced overall size and increased shell in order to boost their optical gain. However, for these type-I QDs where most of the process of recombination takes place in the core area, the reduced core size may lead to poorer lasing performance compared to the previous one with lager core size. It would be more persuasive if the author could provide the information of stimulated emission of this kind of materials and make a compassion of these two.

2). In the second part ’Lasing in LED-like device stacks’, the authors investigate the optimal thickness of ETL and QDs layer for achieving better lasing performance. However, the final fabricated structure shows much higher threshold at 65.6 µJcm-2 due to the spectral shift of the DFB grating resonances. Since they only change the thickness which affects the effective refractive index of the gain, it is important to consider about altering the grating period to meet the requirements of resonance.

3). For QD lasers, the CW regime is closely similar to the electrical excitation. Therefore, it would be really promising if their device could perform lasing under the CW excitation before they proceed to do the electrical pumping experiment. Here they only show lasing under fs laser pumping. Have the authors tried CW pumping?

4). In the discussion section, the authors propose a way to demonstrate optical gain in this device by using a similar ‘current-focusing’ method applied in the previous paper by introducing a specially-shaped HIL with reduced contact area with active layer. Will this method change the refractive index across the whole device stack and lead to poor confinement?

5). For electrically pumped lasers, a big challenge lies in the injection efficiency. First, the quality of CQD thin films really matters. The authors shall provide more information about the fabrication and characterization of CQD thin films. Second, inefficiency injection may lead to Joule heating and serious temperature increase. In this case, should thermal management be taken into account?

Reviewer #2 (Remarks to the Author):

The manuscript of Dr. Klimov and colleagues reports on a new type of colloidal quantum dot (QD)

device that can operate as both an optically pumped laser and an electrically-excited LED-like electroluminescent (EL) structure. This is an important piece of work, and a critical step for the creation of laser diodes based on chemically synthesized QDs.

There are several essential advances reported in this paper that have made this demonstration possible. One is a new device architecture that allows the incorporation of a distributed feedback (DFB) resonator into an electron-injecting indium tin oxide (ITO) layer without obstructing the current flow and, at the same time, avoiding emission quenching by the conductive electrode. The next key accomplishment is the demonstration of lasing with an ultrathin active layer which comprises only 3 dot monolayers. Such thin layers can be excited not only optically, but also electrically, as is demonstrated directly in this work by showing good EL performance for these devices. These advances would not be possible without highly engineered QDs that have been carefully crafted to produce high emission efficiencies for both single-exciton and multi-exciton species. The nonradiative Auger processes are suppressed in these QDs. The latter is very important for lasing applications, as optical gain in QDs relies on emission from multi-excitons.

Overall, this is a well-written manuscript containing novel, high-quality technical materials. The reported research is very important because the development of solution-processable laser diodes is important for many different disciplines due to the wide scope of their potential applications. I strongly recommend this paper for publication in Nature Communications. Below I provide a list of suggestions and questions for clarification, as well as optional revisions.

(1) Why is the emission QY of the QDs only 80%? Can it be improved?

(2) Commonly, semiconductor laser diodes utilize a vertical cavity design. Why did the authors pursue a less common DFB architecture? Also, why do they use the 2nd order DFB grating but not the 1st order?

(3) What is the purpose of the ZnO layer on top of the ITO electrode? Why not deposit QDs directly onto ITO?

(3) The authors state that in order to achieve population inversion they need to increase current density to ~3-4 A/cm^2. This seems to be too low by the standards of existing laser diodes which normally require 10s and 100s of A/cm^2 pumping. Why is the projected threshold current so low?

(4) What is the plan to reach the levels of electrical pumping sufficient for lasing? Will it feasible with the existing configuration or will it require a major re-design of the device structure?

Response to Reviewer 1

The manuscript titled "Optically Pumped Colloidal-Quantum-Dot Lasing in LED-Like Devices with an Integrated Optical Cavity" reports the demonstration of the lasing performance of ultrathin devices made of CdSe based colloidal quantum dots (QD) incorporated into LED configurations with the conductive ITO electrode serving as the grating plane for a DFB laser cavity. The authors start with discussion of challenges needed to be overcome to achieve true electrically pumped lasing from colloidal quantum dots, then demonstrate a continuously graded (cg) QDs with the DFB resonator embedded into an ITO cathode of an inverted QD LED and explain how this specific device could fulfill the requirements to solve the above challenges. From simulation and experimental results, they claim that LED-like device could behave as good single mode lasers with reduced mode leakage. Later, the authors achieved EL with fairly good EQE based on the same LED-like device with DFB resonator. Although this work did not realize electrically pumped lasing from colloidal quantum dots, there are substantial advances in important aspects underlying electrically pumped colloidal lasers, such as cavity platform, effects of electrodes on gain medium, electrical injection etc. Over all, this work will help researchers to understand the key factors for achieving electrically-pumped lasing and will inspire them to fabricate new photonic devices. Therefore, I suggest to accept this manuscript if the authors are able to address the following issues:

We would like to thank the Reviewer for strongly supporting our work and recommending it for publication.

1. In the first part of result section, the author proposed to use the similar dots applied in previous reports but with reduced overall size and increased shell in order to boost their optical gain. However, for these type-I QDs where most of the process of recombination takes place in the core area, the reduced core size may lead to poorer lasing performance compared to the previous one with lager core size. It would be more persuasive if the author could provide the information of stimulated emission of this kind of materials and make a compassion of these two.

We would like to clarify the distinctions between the cg-QD samples used in the present study and those from our previous report of optical gain under direct current (d.c.) electrical pumping [Lim, et al., Nat. Mater. **17**, 42, 2018]. Importantly, in both cases, the CdSe core size was the same (r_{core} = 2 nm), implying that these two samples exhibited similar degrees of spatial confinement. What is distinct between the samples is the thickness of an intermediate compositionally graded $Cd_xZn_{1-x}Se$ layer and a final protective $ZnSe_xS_{1-x}$ shell (see Table R1 below for dimensions of various elements of the two cg-QD samples). In particular, the overall diameter of the dots from the present study is ~3 nm smaller than that from the previous publication. Therefore, while exhibiting a comparable degree of suppression of Auger decay, the new dots allow for considerably higher packing density (by ~60% in terms of the semiconductor volume fraction), which leads to enhanced optical gain and, as a result, reduced lasing thresholds. Indeed, as we show in our manuscript, the DFB devices utilizing these smaller-size cg-QDs demonstrate strong lasing with

pump powers that correspond to average QD excitonic occupancies of >1.1, suggesting that lasing threshold in these structures is near the 'ideal' single-exciton per-dot limit (Fig. 1e).

Table R1. Comparison of dimensions of the cg-QDs used in the present study and those from the previous report (Lim, et al., Nat. Mater. **17**, 42, 2018).

	Present study	Previous study (Lim, et al., Nat. Mater. **17**, 42, 2018)
CdSe core radius	2 nm	2 nm
$Cd_xZn_{1-x}Se$ layer thickness	4 nm	7 nm
Protective layer thickness	1.8 nm	0.4 nm
QD diameter	15.6 nm	18.6 nm
Lasing threshold	$\langle N_{las} \rangle = 1.1$ in QD/DFB lasing	$\langle N_{las} \rangle = 1.6$ in random lasing

2. In the second part 'Lasing in LED-like device stacks', the authors investigate the optimal thickness of ETL and QDs layer for achieving better lasing performance. However, the final fabricated structure shows much higher threshold at 65.6 μJcm-2 due to the spectral shift of the DFB grating resonances. Since they only change the thickness which affects the effective refractive index of the gain, it is important to consider about altering the grating period to meet the requirements of resonance.

We agree with the Reviewer's comment on the importance of spectral matching between the QD gain band and the DFB resonance. As stated in the main text, the Bragg resonance in our 2nd-order QD/DFB cavity is given by $\lambda_{2B} = n_{eff}\Lambda$ and thus depends on both the effective refractive index (n_{eff}) and the grating period (Λ). For example, a small change in n_{eff} by just 0.1 leads to a large spectral shift of the Bragg resonance by $\Delta\lambda_{2B} = 35$ nm. Presently, there is no effective ways for controlling n_{eff} with sub-0.1 accuracy due to a multitude of parameters that affect n_{eff} including the QD packing density, the amounts of bound and unbound ligands, the QD-film thickness, etc. Therefore, presently we adjust a grating period for achieving the spectral match between the Bragg resonance and the gain band. However, due to unpredictable variations in n_{eff}, this approach is extremely labor intensive and time consuming as we fabricate DFB gratings one by one. Presently, we are working on an 'accelerated testing' approach that will allow for fabricating multiple gratings with a varied period on the same chip. This project is in progress and upon the completion it will be applied to improve matching between λ_{2B} and the QD gain band.

3. For QD lasers, the CW regime is closely similar to the electrical excitation. Therefore, it would be really promising if their device could perform lasing under the CW excitation before they proceed to do the electrical pumping experiment. Here they only show lasing under fs laser pumping. Have the authors tried CW pumping?

Indeed, the regime of QD lasing under cw-optical pumping is, in principle, similar to that of lasing under d.c. electrical injection. However, there is an important distinction in the amount of heat released in these two cases. In particular, as was demonstrated in ref [Lim, et al., Nat. Mater. **17**, 42, 2018], optical gain could be realized with d.c. electrical pumping with minimal overheating of the QD emitting layer by just ~30 K. On the other hand, the amount of excess heat released during cw optical pumping is much greater. In experiments with optical excitation, the pump photon energy ($h\nu_p$) is typically high (~3 eV) in order to take advantage of a large absorption cross-section of a wide-gap shell. As a result, each photogenerated electron-hole pair carries a large amount of excess energy (δE). For example, for the QD band-gap of 2 eV and $h\nu_p = 3$ eV, δE is ~1 eV. This corresponds to an extremely high effective temperature of ~3,800 K. The management of this excessive heat is a difficult engineering problem, which was resolved only recently in the 2017 reports on the first colloidal QD-based cw lasers [Fen, et al., Nature **544**, 75, 2017]. The demonstrated devices employed sophisticated heat-management schemes; however, even in this case, they showed stable performance only for a few minutes and eventually failed due to overheating. Resolving the problem of excess heat generation in our LED-like devices would require a dedicated study, which is outside the scope of the present manuscript (see our response to this Reviewer's comment on heat management later in this document). In fact, per our earlier discussion, in the case of prospective electrically pumped lasers, the problem of heat generation may not be as severe as in optically pumped structures as electrically injected charges do not carry a large amount of excess energy.

4. In the discussion section, the authors propose a way to demonstrate optical gain in this device by using a similar 'current-focusing' method applied in the previous paper by introducing a specially-shaped HIL with reduced contact area with active layer. Will this method change the refractive index across the whole device stack and lead to poor confinement?

In current-focusing LED structures demonstrated in our previous study [Lim, et al., Nat. Mater. **17**, 42, 2018], we used a stripe-like hole injection layer which was 70 - 100 μm wide and 1.5 mm long. This size of the active area is sufficient for maintaining a stable lasing action. In fact, as we demonstrate in the present manuscript, it is possible to realize strong lasing even with a smaller area defined by the cross-section size of the focused pump beam of just ~30×390 μm^2 (see inset of Supplementary Fig. 3c and Fig. R1 below). Therefore, we do not expect that the use of 'current focusing' will impede lasing action in future electrically pumped lasing devices.

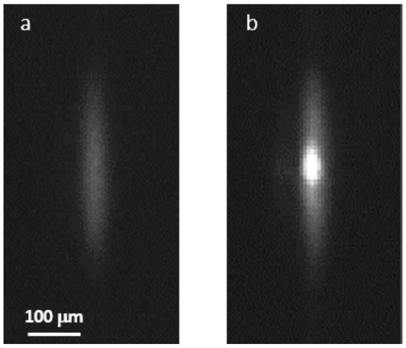

Figure R1. Spatial profiles of a stripe-shaped pump beam (~30×390 µm^2 area) focused onto a QD/DFB device before (a) and after (b) the lasing threshold. The bright spot in the middle of the excited device area is emission in the direction normal to the DFB grating due to out-of-plane 1-st order scattering.

5-1. For electrically pumped lasers, a big challenge lies in the injection efficiency. First, the quality of CQD thin films really matters. The authors shall provide more information about the fabrication and characterization of CQD thin films.

In response to this comment, we have conducted additional characterization of the effective refractive index of QD films ($n_{\mathrm{eff,TE}}$) and performed the analysis of statistical device-to-device variations in $n_{\mathrm{eff,TE}}$ as well as their implications for lasing performance. The results of these studies are incorporated in the revised Supplementary Fig. 3 as a new panel 'f'. Their discussion is included in Methods in the expanded section "Fabrication of QD DFB lasers." It reads (pp. 16-17):

> **Fabricated devices usually showed lasing into a transverse electrical (TE) mode (see Supplementary Fig. 3d,e). The effective refractive index of the QD films ($n_{\mathrm{eff,TE}}$) was determined from the expression for the 2nd order Bragg scattering using the known DFB grating period and the measured position of the center of the photonic gap observed for the TE polarization. Based on the measurements of 17 devices, the average value of $n_{\mathrm{eff,TE}}$ was 1.76. A device-to-device variation in $n_{\mathrm{eff,TE}}$ was within ± 0.05 (Supplementary Fig. 3f). Despite a fairly small uncertainty in the refractive index (< 3%), the resulting variation in λ_{2B} was quite large (±18 nm). Therefore, for each type of a multilayered device, we fabricated several gratings with different periods (see previous section) and upon completion of a device stack, we characterized their Bragg patterns. For lasing experiments, we selected samples which showed the closest match between the Bragg TE mode and the optical gain band. In the case of devices with especially good spectral matching, we were able to achieve lasing thresholds which were near the fundamental limit of one electron-hole pair per dot on average (see, e.g., Fig. 1e).**

5-2. Second, inefficiency injection may lead to Joule heating and serious temperature increase. In this case, should thermal management be taken into account?

Earlier in our response to this Reviewer, we have already had a brief discussion of heat management in QD lasers. As we indicated in this discussion, the problem of excess heat generation is likely less severe in the case of electrical pumping compared to the situation of optical pumping employing photons of high energy. Nonetheless, if required, we are ready to apply several strategies for managing heat in high-current-density QD-LEDs. One involves the use of pulsed 'quasi-d.c.' electrical pumping which imitates pulsed optical excitation and allows for reducing heat generation by lowering the pulse repetition rate. In addition, we will apply a 'device-level' strategy for heat management which will involve incorporation of high-thermal conductivity ($\sigma_{thermal}$) elements in a device stack. For examples, the replacement of a glass substrate ($\sigma_{thermal}$ = 1.4 W m^{-1} K^{-1}) with a magnesium fluoride substrate ($\sigma_{thermal}$ > 20 W m^{-1} K^{-1}) should help dissipate heat out of the QD layer [see, e.g., Adachi, et al., Nat. Comm. **6**, 8694, 2015]. Furthermore, for implementing a current-focusing architecture, we plan to use a dielectric aperture made of lithium fluoride (LiF) for both guiding the current towards the emitting layer and also for improving heat dissipation as LiF exhibits high thermal conductivity ($\sigma_{thermal}$ > 10 W m^{-1} K^{-1}).

Response to Reviewer 2

The manuscript of Dr. Klimov and colleagues reports on a new type of colloidal quantum dot (QD) device that can operate as both an optically pumped laser and an electrically-excited LED-like electroluminescent (EL) structure. This is an important piece of work, and a critical step for the creation of laser diodes based on chemically synthesized QDs. There are several essential advances reported in this paper that have made this demonstration possible. One is a new device architecture that allows the incorporation of a distributed feedback (DFB) resonator into an electron injecting indium tin oxide (ITO) layer without obstructing the current flow and, at the same time, avoiding emission quenching by the conductive electrode. The next key accomplishment is the demonstration of lasing with an ultrathin active layer which comprises only 3 dot monolayers. Such thin layers can be excited not only optically, but also electrically, as is demonstrated directly in this work by showing good EL performance for these devices. These advances would not be possible without highly engineered QDs that have been carefully crafted to produce high emission efficiencies for both single-exciton and multi-exciton species. The nonradiative Auger processes are suppressed in these QDs. The latter is very important for lasing applications, as optical gain in QDs relies on emission from multi-excitons.

Overall, this is a well-written manuscript containing novel, high-quality technical materials. The reported research is very important because the development of solution-processable laser diodes is important for many different disciplines due to the wide scope of their potential applications. I strongly recommend this paper for publication in Nature Communications.

We would like to thank the Reviewer for strongly supporting our work and recommending it for publication.

Below I provide a list of suggestions and questions for clarification, as well as optional revisions.

1. Why is the emission QY of the QDs only 80%? Can it be improved?

Yes, the PL QY of cg-QDs can be improved to values beyond 80%. A primary reason for a nonideal PL efficiency is defects at the core/shell interface and imperfections in the shell region. One source of such defects are impurities in precursors that are incorporated into the QDs during the growth process. Presently, we are working on developing approaches for thorough precursor purification prior to their use in the QD synthesis. We have already gotten first successful results with this approach. In particular, the most recent generation of cg-QDs prepared using "in-house" purified precursors show PL QYs of ~90% and even higher. This work is in progress and eventually will be a subject of a separate manuscript.

2. Commonly, semiconductor laser diodes utilize a vertical cavity design. Why did the authors pursue a less common DFB architecture?

There are several reasons for selecting a DFB cavity versus a vertical cavity. In particular, a DFB grating can be readily integrated into a QD-LED without altering existing architectures optimized for high efficiency carrier injection. In addition, the DFB cavity resonances can be easily adjusted by changing the lateral periodicity without modifying the vertical structure of a multilayered device. Further, the DFB cavity provides high single-pass gain due to multiple coherent Bragg scattering events in the plane of the grating.

Also, why do they use the 2nd order DFB grating but not the 1st order?

The primary reason for using the 2nd but not the 1st order grating is resolution limits of laser interferometric lithography used in our work. In order to match the 2nd order Bragg resonance with the gain band of our QD samples, we use grating periods of 360 to 380 nm. Such structures can be easily defined using photolithography. The situation is different in the case of the 1st order cavities that would require gratings with a period which is twice as short, i.e., is in the range of 180-190 nm. Such feature sizes are not accessible with laser interferometry and would require much more expensive and "slow" e-beam lithography.

In addition to being more accessible from the fabrication standpoint, the 2nd order grating offers certain advantages from the device-characterization standpoint. While, in-plane optical feedback in this case is realized due to 2nd order Bragg scattering, the 1st order scattering leads to fairly intense out-of-plain surface emission. This provides a convenient way to characterize photonic properties of the DFB cavity (e.g., spectral structure, polarization, and dispersion characteristics of photonic modes) and to monitor lasing performance.

3. What is the purpose of the ZnO layer on top of the ITO electrode? Why not deposit QDs directly onto ITO?

ZnO is typically used in state-of-the-art QD-LEDs as an electron transport layer. Its purpose is to spatially separate the QD emitting layer from a highly conductive ITO electrode (a cathode) and thereby prevent emission quenching. In addition, the Fermi level of n-type ZnO closely matches the conduction band of the QDs which facilitates electron injection. Finally, ZnO exhibits good chemical affinity with QD surface ligands which simplifies preparation of a highly uniform, pinhole-free QD emitting layer, which is important for achieving spatially uniform carrier injection and preventing current leakage.

4. The authors state that in order to achieve population inversion they need to increase current density to ~3-4 A/cm^2. This seems to be too low by the standards of existing laser diodes which normally require 10s and 100s of A/cm^2 pumping. Why is the projected threshold current so low?

Indeed, standard laser diodes based on epitaxially-grown QDs (epi-QDs) normally require high current densities of ~100 A cm^{-2}. Record performing epi-QD-based devices, however, demonstrated lasing thresholds as low as ~10 A cm^{-2} [Deppe, et al., Electron. Lett., **45**, 54, 2009]. In the case of QD gain media, the lasing threshold strongly depends on QD size. In particular, the decrease of the QD size is expected to lower the threshold due to the increased energy separation between discrete electronic states which increases the occupancy of the band-edge emitting levels. Colloidal QDs are considerably smaller than epi-QDs, which should decrease the lasing threshold. Indeed, as we demonstrated recently [Lim, et al., Nat. Mater., **17**, 42, 2018], using colloidal QDs we could achieve optical gain with current densities of only 3 - 4 A cm^{-2}. We used these values to project the threshold current density required to obtain lasing in our devices.

5. What is the plan to reach the levels of electrical pumping sufficient for lasing? Will it feasible with the existing configuration or will it require a major re-design of the device structure?

We believe that the present configuration (LED with an integrated DFB cavity) will allow us to realize a true lasing action. Presently, we are about a factor of 10 below the required current density (*j*). In our ongoing effort, we work on increasing *j* by reducing the injection area. This idea of 'current-focusing' was successfully employed in our recent demonstration of optical gain in QD-LEDs [Lim, et al., Nat. Mater. **17**, 42, 2018]. In our present manuscript, we use devices with a relatively large active area of 1,500 μm × 1,500 μm. To implement current focusing, we plan to limit the device area to a rectangle with dimensions of ca. 30 μm x 400 μm. This would allow us to boost the current density by a factor of ~200, which will be sufficient to reach and even exceed the threshold requirement. Our preliminary tests with optical excitation show that we can achieve a stable lasing action with the 30 μm x 400 μm size of the excitation spot. This indicates the feasibility of lasing with electrical excitation for the same size of the active area. The new data on lasing with a small excitation spot size are included in the revised SI as the insets of Supplementary Fig. 3c (see below).

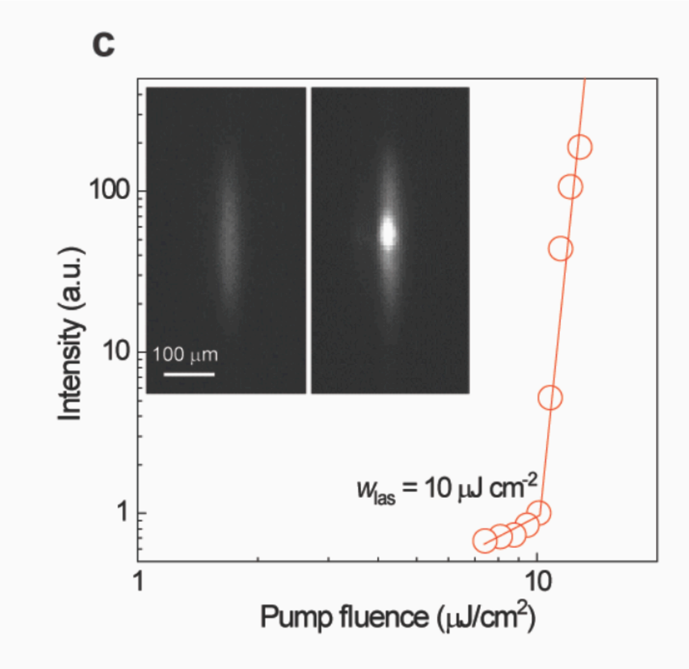

c, Surface emission intensity as a function of pump fluence indicates a sharp lasing threshold (w_{las}) of ~10 μJ cm^{-2}. The insets show spatial profiles of a stripe-shaped pump beam (~30×390 μm^2 area) focused onto a QD/DFB device before (left) and after (right) the lasing threshold. The bright spot in the middle of the excited device area is lasing emission in the direction normal to the DFB grating due to out-of-plane 1st-order scattering.

Summary of Studies for Lasing Reporting Checklist

Lasing reporting summary. We have completed this form and have revised the main text and the Supplementary Information to include information from additional measurements conducted in response to some of the questions on this form. Below, we provide a summary of the corresponding revisions.

In response to **Question #3**, we have conducted studies of polarization of emission from our devices before and after the lasing threshold. The results of these studies are included in the revised Supplementary Fig. 3 (see below).

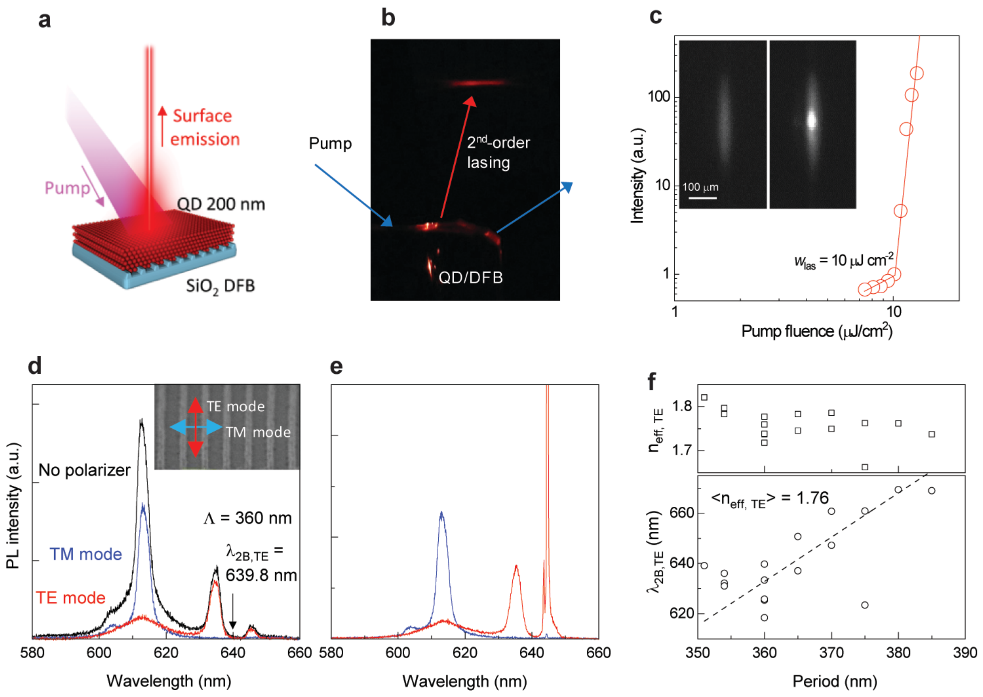

Supplementary Fig. 3| a, A schematic depiction of a QD laser based on the second-order SiO_2 DFB resonator. b, A photograph of an operating device which lases in the direction normal to the DFB surface, as expected for the second-order grating. c, Surface emission intensity as a function of pump fluence indicates a sharp lasing threshold (w_{las}) of ~10 μJ cm^{-2}. The insets show spatial profiles of a stripe-shaped pump beam (~30×390 μm^2 area) focused onto a QD/DFB device before (left) and after (right) the lasing threshold. The bright spot in the middle of the excited device area is lasing emission in the direction normal to the DFB grating due to out-of-plane 1st-order scattering. d, Emission spectra of the QD/DFB cavity before the lasing threshold measured without a polarizer (black) and with a linear polarizer for polarizations along (TE mode; the red arrow in the inset and the red trace in the main panel) and perpendicular (TM mode; the blue arrow in the inset and the blue trace in the main panel) to the DFB grating grooves. e, Polarization-resolved measurements above the lasing threshold (same color coding as in 'd') indicate that lasing occurs into the linear-polarized TE mode. f, The wavelength of the 2nd-order

TE Bragg resonance, $\lambda_{2B,TE}$ (based on the position of the center of the TE stop band; panel 'd'), as a function of grating period, Λ (bottom sub-panel). The effective refractive index ($n_{eff,TE}$) determined from the Bragg condition based on the measured $\lambda_{2B,TE}$ as a function of Λ (top sub-panel). Source data are included as the Source Data file.

In response to **Question #4**, we have included an image of the surface emitted beam above the lasing threshold as Supplementary Fig. 3b (see above). This image illustrates high directionality of the emitted light, as expected for the lasing effect.
In response to **Question #6**, we have expanded Methods section "DFB lasing measurements" to detail our observations in support of the claim of lasing in the fabricated devices (see below).

Below the threshold pump fluence (w_{las}), we observed narrow (~3 nm full width at half maximum, FWHM), linearly-polarized transverse electrical (TE) and transverse magnetic (TM) cavity modes along with a weaker, unpolarized, spectrally broad (~20 nm FWHM) emission. When the pump fluence was raised above w_{las}, a sharp spectral line (~0.2 nm FWHM) emerged on top of one of the cavity modes (typically, a lower-energy TE peak). The intensity of this line exhibited very fast, super-linear growth with increasing pump level characterised by a log-log slope of 10–20 or more. The outcoupled light was highly directional and propagated in the direction normal to the DFB surface, as expected for the second-order DFB cavity (Supplementary Fig. 3 b,c). All of these observations were consistent with the lasing action in a QD/DFB cavity and could not be explained by alternative processes such as amplified spontaneous emission, directional scattering, or cavity modified fluorescence. The first of these effects would not be linked to cavity resonances, while the other two would not exhibit a well-defined threshold behaviour.

In response to **Question #7**, we have revised the manuscript to include the reference (#38) which contains theoretical modelling of the optical gain threshold in QD samples similar to those used in the present study. The corresponding text reads (page 8):

The measured onset of the lasing effect is only ~5.5 μJ cm^{-2}, which is comparable to the lowest values previously reported for any type of QD lasers[11]. The corresponding average per-dot excitonic occupancy, $\langle N_{las} \rangle$, is 1.1 (see Methods). This value is close to the theoretical gain threshold calculated for cg-QD in ref 38 suggesting that the use of L-ITO as a DFB grating material indeed allows for realizing high-quality, low-loss optical cavities.

In response to **Question #8**, we have prepared a summary of lasing thresholds for all studied devices and included it in the revised manuscript as Supplementary Table 1 (see below).

DFB material	Device structure	Lasing threshold (μJ cm^{-2})	Figure
SiO_2	DFB/QD100	13.8	3b
	DFB/QD100	24.3	
	DFB/QD150	8.0	3b
	DFB/QD200	10	3b

	DFB/QD200	14.5	
	DFB/QD200	21	
	DFB/QD300	6.0	3b
	DFB/QD50/TCTA60	18.8	3c
	DFB/QD100/TCTA50	12.9	3b
	DFB/QD100/TCTA85	13.3	3b
	DFB/QD100/TCTA145	14.5	3b
	DFB/QD100/TCTA270	13.3	3b
	DFB/QD100/TCTA500	18.5	3b
	DFB/QD150/TCTA60	11.5	
	DFB/QD150/TCTA60	19.6	
	DFB/QD200/TCTA60	24.5	
	DFB/QD200/TCTA60	28	
	DFB/QD200/TCTA60	30.8	
L-ITO	DFB/QD200	13	
	DFB/QD200	15	
	DFB/QD250	5.5	1e
	DFB/QD250	18	
	DFB/QD200/TCTA60	17	
	DFB/ZnO50/QD200	8.1	
	DFB/ZnO50/QD250	8.5	
	DFB/ZnO50/QD250	5.7	2c
	DFB/ZnO50/QD250/TCTA60	17	2c
	DFB/ZnO50/QD50/TCTA60	65	4c

Supplementary Table 1| The summary of lasing thresholds observed for 28 devices fabricated and tested in the present study. The number, which appears in the 2nd column next to the type of the specific device layer (QD, ZnO, or TCTA) indicates its thickness in units of nm. During a given test, the fabricated devices showed stable performance on a time scale of over 10 hours of continuous operation. This performance was reproducible for several days.

REVIEWERS' COMMENTS:

Reviewer #1 (Remarks to the Author):

Developing electrically pumped lasers is a very significant task, and remains challenging. Now effort toward this direction is at the critical stage. This manuscript offers useful insights and represent substantial progress in this field, although true optical injected lasing has not been realized. After carefully examing the revised version and the response to the comments, I believe that it's publishable.

Handong Sun

Reviewer #2 (Remarks to the Author):

I am completely satisfied with the answers of the authors to my questions. I also believe the authors have performed a tremendous amount of work answering on the firts referees comments, suggestions and criticisms. I am also impressed by the data reported by Klimov group for the lasing reporting checklist. These data remain no doubt that the authors observed lasing. I strongly recommend the paper publication in Nature Communication magazine.
